# Lipid Metabolic Reprogramming and Bioactive Lipid Signaling in MASLD: Molecular Mechanisms, Pathogenesis and Therapeutic Opportunities

**DOI:** 10.3390/ijms27177805

**Published:** 2026-08-31

**Authors:** Tatjana Ábel, Éva Csobod Csajbókné

**Affiliations:** Department of Dietetics and Nutritional Sciences, Faculty of Health Sciences, Semmelweis University, 1088 Budapest, Hungary

**Keywords:** MASLD, MASH, lipid metabolic reprogramming, lipotoxicity, lipid signaling, ceramides, lipidomics, mitochondrial dysfunction, fibrosis, precision hepatology

## Abstract

Metabolic dysfunction-associated steatotic liver disease (MASLD) is increasingly recognized as a disorder of hepatic lipid metabolic reprogramming rather than a consequence of passive triglyceride accumulation. Chronic nutrient excess, insulin resistance, adipose tissue dysfunction, and altered nutrient-sensing pathways disrupt the balance among hepatic fatty acid uptake, de novo lipogenesis, β-oxidation, lipid storage, and lipoprotein export. These changes promote the accumulation of bioactive lipid species, including saturated fatty acids, ceramides, diacylglycerols, oxidized phospholipids, and free cholesterol. Unlike triglycerides, which may serve an adaptive buffering role under conditions of preserved lipid-storage capacity, these bioactive lipids function as metabolic stress signals that impair insulin signaling, disrupt mitochondrial and endoplasmic reticulum homeostasis, activate PKC, JNK, MAPK, NF-κB, and NLRP3 pathways, and promote hepatocyte death, immune activation, and fibrogenesis. Emerging lipidomic and multi-omic approaches further demonstrate that the molecular composition and subcellular distribution of hepatic lipids may be more closely associated with disease progression than total lipid content. This review critically integrates current evidence on hepatic lipid metabolic reprogramming, lipid-mediated signaling, organelle dysfunction, fibrosis, and molecular heterogeneity in MASLD. It also evaluates the translational potential and limitations of lipidomic biomarkers and mechanism-based therapies targeting lipogenesis, nuclear receptors, ceramide metabolism, inflammatory signaling, and fibrosis. A deeper understanding of disease-specific lipid signatures and signaling networks may support molecular endotyping, mechanism-based therapeutic strategies, and precision hepatology in patients with metabolic dysfunction-associated steatohepatitis (MASH) and progressive fibrosis.

## 1. Introduction

Metabolic dysfunction-associated steatotic liver disease (MASLD) has become the most prevalent chronic liver disease worldwide and represents a major public health challenge because of its close association with obesity, insulin resistance, type 2 diabetes mellitus (T2DM), dyslipidemia, and cardiovascular disease [1,2,3,4]. The global prevalence of MASLD continues to rise in parallel with the increasing burden of metabolic disorders, affecting approximately one-third of the adult population worldwide [5,6]. Beyond liver-related morbidity and mortality, MASLD substantially increases the risk of cardiovascular disease, chronic kidney disease, hepatocellular carcinoma (HCC), and overall mortality, placing a considerable socioeconomic burden on healthcare systems [2,3,7].

The recently adopted nomenclature replacing nonalcoholic fatty liver disease (NAFLD) with MASLD reflects a major conceptual shift in disease classification. In 2023, an international multi-society Delphi consensus proposed the new terminology to emphasize metabolic dysfunction as the principal driver of disease development rather than defining the condition by the exclusion of alcohol consumption [8]. Within this framework, metabolic dysfunction-associated steatohepatitis (MASH) designates the progressive inflammatory phenotype characterized by hepatocellular injury, inflammation, and varying degrees of fibrosis [8,9].

Historically, hepatic steatosis was regarded as a relatively benign consequence of excessive triglyceride accumulation. However, triglyceride sequestration within lipid droplets is generally less cytotoxic than the accumulation of non-esterified fatty acids and bioactive lipid intermediates and may initially function as an adaptive buffering mechanism. Disease progression is more closely associated with the excessive formation and redistribution of bioactive lipid species that activate cellular stress responses collectively referred to as lipotoxicity [10,11,12,13,14]. Saturated fatty acids, ceramides, sphingolipids, diacylglycerols, oxidized phospholipids, bile acids, and free cholesterol disrupt mitochondrial function, induce endoplasmic reticulum stress, promote oxidative injury, activate inflammatory signaling pathways, and stimulate fibrogenesis, thereby linking metabolic overload to progressive liver damage [11,12,13,14,15,16,17,18,19,20,21,22,23,24]. Consequently, MASLD is now recognized as a disorder of dysregulated lipid homeostasis rather than simple triglyceride accumulation.

A central feature of MASLD pathogenesis is hepatic lipid metabolic reprogramming, whereby chronic nutrient excess and insulin resistance reshape lipid handling through increased adipose tissue lipolysis, enhanced de novo lipogenesis, impaired fatty acid β-oxidation, defective very-low-density lipoprotein (VLDL) secretion, and altered cholesterol metabolism [12,15,16,17]. These metabolic alterations are orchestrated by interconnected nutrient-sensing pathways and transcriptional regulators, including sterol regulatory element-binding protein-1c (SREBP-1c), carbohydrate-responsive element-binding protein (ChREBP), AMP-activated protein kinase (AMPK), mechanistic target of rapamycin (mTOR), peroxisome proliferator-activated receptors (PPARs), farnesoid X receptor (FXR), and liver X receptor (LXR), which collectively determine the balance between lipid synthesis, storage, oxidation, and export [15,16,17,18,19]. Rather than representing a secondary consequence of steatosis, lipid metabolic reprogramming actively shapes disease evolution by continuously remodeling intracellular signaling networks and organelle function.

Beyond their metabolic functions, lipids have emerged as potent signaling molecules that regulate numerous cellular processes involved in disease progression. Lipotoxic lipid species activate signaling cascades such as protein kinase C (PKC), c-Jun N-terminal kinase (JNK), mitogen-activated protein kinase (MAPK), nuclear factor-κB (NF-κB), and the NLRP3 inflammasome, thereby integrating metabolic stress with inflammation, hepatocyte death, immune activation, and extracellular matrix remodeling [20,21,22,23,24]. In parallel, lipid-induced dysfunction of mitochondria, the endoplasmic reticulum, lysosomes, and autophagic pathways further amplifies oxidative stress and fibrogenic responses, creating self-perpetuating mechanisms that drive progression from simple steatosis to MASH and advanced fibrosis.

Recent advances in lipidomics, metabolomics, proteomics, single-cell transcriptomics, and systems biology have substantially expanded our understanding of the molecular complexity of MASLD [25,26,27,28,29]. These technologies have identified disease-specific lipid signatures associated with disease severity, fibrosis progression, and therapeutic responsiveness, providing new opportunities for biomarker discovery and precision hepatology while simultaneously revealing novel mechanism-based therapeutic targets.

This review provides an integrated and critically evaluated framework linking hepatic lipid metabolic reprogramming to lipid-mediated signaling, organelle dysfunction, immune activation, and fibrogenesis in MASLD. In contrast to approaches that consider these processes separately, we emphasize that disease progression is determined not only by the amount of hepatic lipid accumulation but also by the molecular identity, subcellular localization, and signaling activity of individual lipid species. We further examine how these interconnected mechanisms may define distinct molecular endotypes of MASLD and influence biomarker performance and therapeutic responsiveness. By integrating mechanistic, lipidomic, and translational evidence, this review aims to identify both established pathogenic nodes and unresolved questions that should guide future precision-based therapeutic strategies. Unlike previous reviews that primarily address individual lipid species, signaling pathways, or metabolic processes, the present review integrates lipid metabolic reprogramming, bioactive lipid signaling, organelle dysfunction, lipidomics, molecular endotypes, and emerging therapeutic strategies into a unified mechanistic framework linking lipid dysregulation to disease progression.

## 2. Literature Search Strategy

This structured narrative review was based on a literature search of PubMed, Scopus, and Web of Science for English-language publications addressing lipid metabolism, lipid-mediated signaling, lipotoxicity, lipidomics, fibrosis, and therapeutic development in MASLD and MASH. Search terms included combinations of “MASLD”, “MASH”, “NAFLD”, “NASH”, “lipid metabolism”, “lipotoxicity”, “ceramide”, “sphingolipid”, “diacylglycerol”, “lipid signaling”, “lipidomics”, “mitochondrial dysfunction”, “endoplasmic reticulum stress”, “fibrosis”, and “therapeutic target”. Representative search combinations included (“MASLD” OR “MASH” OR “NAFLD” OR “NASH”) AND (“lipid metabolism” OR “lipotoxicity” OR “lipid signaling”), (“MASLD” OR “MASH”) AND (“ceramide” OR “sphingolipid” OR “diacylglycerol”), and (“MASLD” OR “MASH”) AND (“lipidomics” OR “mitochondrial dysfunction” OR “endoplasmic reticulum stress” OR “fibrosis”).

Studies were considered eligible if they addressed mechanisms of hepatic lipid metabolic reprogramming, bioactive lipid signaling, lipotoxicity, organelle dysfunction, inflammation, fibrosis, lipidomic biomarkers, or mechanism-based therapeutic strategies relevant to MASLD/MASH. Original mechanistic studies, human observational studies, clinical trials, consensus statements, systematic reviews, and high-quality recent reviews were prioritized. Seminal older studies were retained when necessary to establish fundamental mechanistic concepts. Studies were excluded if they were not directly relevant to the predefined scope, lacked sufficient mechanistic or clinical relevance to MASLD/MASH, were available only as abstracts without adequate methodological information, or represented duplicate publications. Non-English-language publications were not included.

The searches covered the literature from database inception through July 2026, with emphasis on recent publications while retaining seminal older studies where necessary to establish fundamental mechanistic concepts. Retrieved publications were screened for relevance to the predefined thematic scope based on titles and abstracts, followed by full-text assessment of potentially relevant articles. Study selection was guided by relevance to the mechanistic and translational objectives of the review, methodological quality, recency, and contribution to areas of uncertainty or conflicting evidence. Reference lists of selected publications were additionally screened to identify relevant studies not captured by the database searches. Given the structured narrative design of this review, no formal systematic quality or risk-of-bias assessment using standardized tools was performed. The final literature search was performed in July 2026.

## 3. Hepatic Lipid Metabolic Reprogramming in MASLD

**Figure 1:**
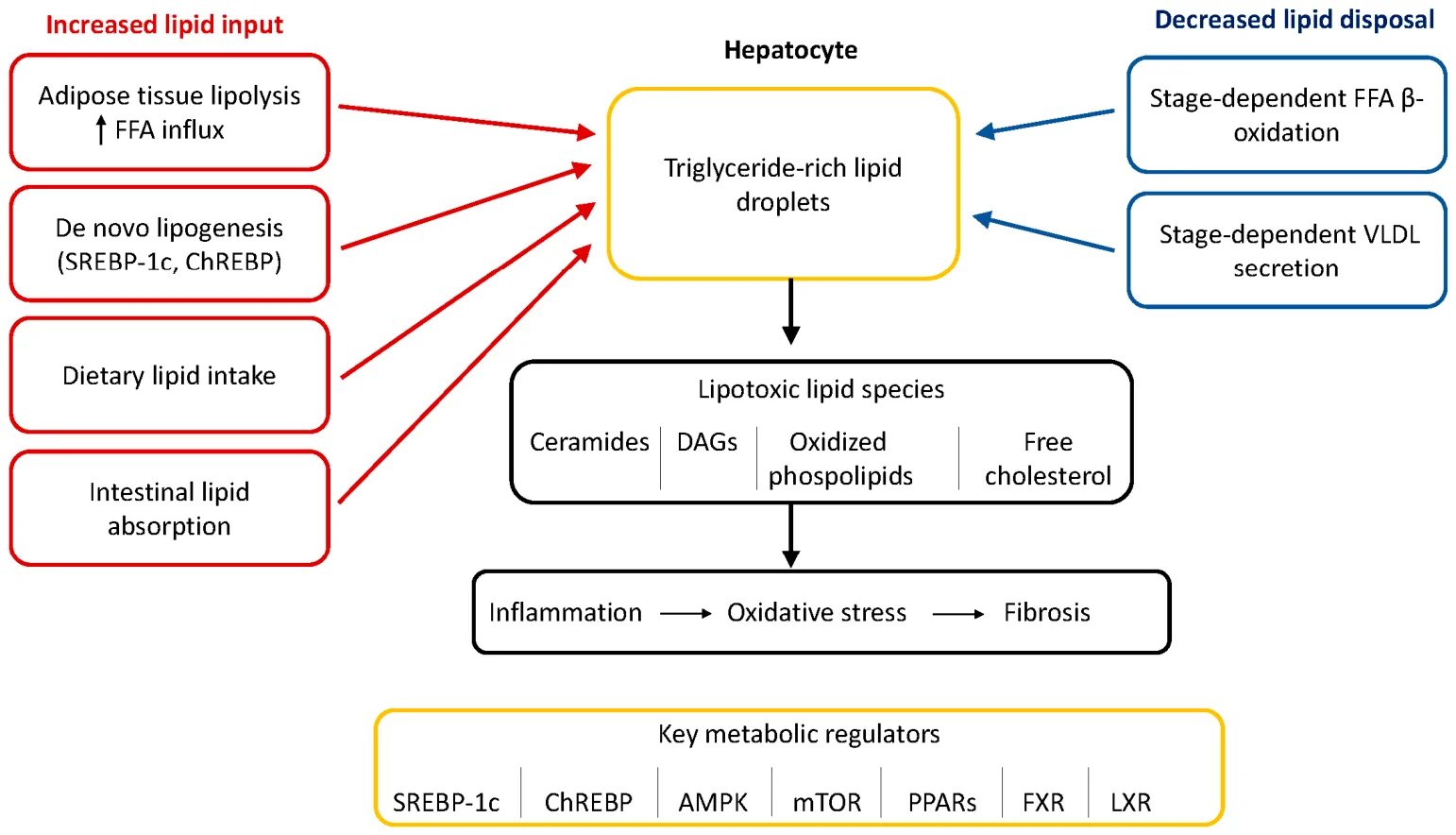
Metabolic pathways driving hepatic lipid accumulation and lipotoxicity in MASLD. Schematic overview of the major metabolic pathways contributing to hepatic lipid accumulation, illustrating the imbalance between increased lipid input and impaired lipid disposal. The resulting accumulation of bioactive lipotoxic lipid species activates oxidative stress, inflammation, and fibrogenic pathways, while nutrient- and energy-sensing regulators contribute to metabolic reprogramming and disease progression. These processes are dynamic and heterogeneous and do not imply a uniform linear progression across all patients with MASLD.

Hepatic lipid accumulation in MASLD results from coordinated metabolic reprogramming rather than dysregulation of a single metabolic pathway. Under physiological conditions, hepatic lipid homeostasis is maintained through a dynamic balance among fatty acid uptake, de novo lipogenesis, mitochondrial β-oxidation, lipid storage, and VLDL secretion [3,11,12,15,16]. Chronic nutrient excess, insulin resistance, adipose tissue dysfunction, and systemic inflammation disrupt this balance, resulting in excessive intrahepatic lipid accumulation and the generation of lipotoxic lipid species that contribute to progressive liver injury [3,11,12,15,16]. The following sections summarize the principal metabolic pathways responsible for hepatic lipid overload and the regulatory mechanisms underlying their dysregulation in MASLD (Figure 1) [15,16,23].

Genetic susceptibility may further modify hepatic lipid metabolic reprogramming and contribute to interindividual heterogeneity in MASLD. Genetic risk variants, including PNPLA3, TM6SF2, MBOAT7, and HSD17B13, have been associated with differences in hepatic lipid metabolism and susceptibility to disease progression, supporting an interaction between inherited predisposition and metabolic drivers of MASLD [12,15,23].

### 3.1. Sources of Hepatic Lipid Accumulation

#### 3.1.1. Adipose Tissue Lipolysis and Free Fatty Acid Flux

Hepatic lipid accumulation results from the imbalance between lipid input and lipid disposal pathways. Adipose tissue-derived free fatty acids (FFAs) constitute the major source of hepatic lipid accumulation in MASLD. Stable isotope tracer studies indicate that approximately 60% of intrahepatic triglycerides originate from adipose tissue lipolysis, whereas de novo lipogenesis and dietary lipid uptake contribute substantially smaller proportions [15,16,30]. Consequently, adipose tissue dysfunction is now recognized as a primary driver of hepatic lipid metabolic reprogramming rather than merely a consequence of systemic metabolic disease.

Under physiological conditions, insulin suppresses adipose tissue lipolysis through inhibition of adipose triglyceride lipase (ATGL) and hormone-sensitive lipase (HSL), thereby limiting the release of non-esterified fatty acids into the circulation. However, obesity and insulin resistance impair this regulatory mechanism, resulting in chronically elevated FFA flux from adipose tissue to the liver despite hyperinsulinemia [12,15,30]. In parallel, visceral adipose tissue undergoes hypertrophy, macrophage infiltration, and chronic low-grade inflammation, leading to increased secretion of pro-inflammatory cytokines and reduced adiponectin production, which further aggravate systemic insulin resistance and hepatic lipid overload [11,30,31].

The continuous oversupply of FFAs exceeds the metabolic capacity of hepatocytes, promoting excessive lipid storage and profound alterations in intracellular lipid metabolism. Hepatic fatty acid uptake is facilitated by transport proteins such as CD36 and fatty acid transport proteins (FATPs), whose expression is increased in MASLD [15,23]. Although esterification into triglycerides initially protects hepatocytes from lipid toxicity, sustained fatty acid influx favors the generation of bioactive lipid intermediates, including ceramides, diacylglycerols, and oxidized lipid species, which contribute to metabolic dysfunction and progressive liver injury [11,13,15].

Importantly, the biological effects of FFAs depend not only on their quantity but also on their composition. Saturated fatty acids, particularly palmitate, induce endoplasmic reticulum stress, mitochondrial dysfunction, oxidative stress, and inflammatory responses more efficiently than monounsaturated fatty acids, thereby accelerating hepatocellular injury and disease progression [11,12,13]. Beyond serving as metabolic substrates, excessive FFAs also initiate intracellular signaling cascades that impair insulin signaling and amplify inflammatory responses, providing a mechanistic link between adipose tissue dysfunction and the development of MASH [18,23].

Collectively, increased adipose tissue lipolysis represents the predominant source of hepatic lipid accumulation in MASLD and constitutes an early event in hepatic lipid metabolic reprogramming. Persistent FFA overload not only supplies excess lipid substrate but also initiates lipotoxic signaling pathways that drive inflammation, fibrosis, and disease progression, as discussed in the following sections.

#### 3.1.2. Enhanced De Novo Lipogenesis

Enhanced hepatic de novo lipogenesis (DNL) is a hallmark of metabolic reprogramming in MASLD and represents the second major source of intrahepatic lipid accumulation after adipose tissue-derived free fatty acids. Under physiological conditions, DNL contributes only modestly to hepatic triglyceride synthesis. However, chronic caloric excess, hyperinsulinemia, and excessive carbohydrate intake, particularly fructose consumption, markedly stimulate this pathway, increasing the conversion of glucose-derived carbon into fatty acids (Figure 1) [15,16,32].

The DNL pathway is initiated by the conversion of citrate-derived acetyl-CoA into malonyl-CoA by acetyl-CoA carboxylase (ACC), followed by fatty acid synthesis catalyzed by fatty acid synthase (FASN) and subsequent desaturation by stearoyl-CoA desaturase-1 (SCD1). These newly synthesized fatty acids are preferentially esterified into triglycerides or incorporated into complex lipid species that contribute to hepatic steatosis and lipotoxicity [15,16,23,32]. Stable isotope studies have demonstrated that hepatic DNL accounts for approximately 25–30% of intrahepatic triglycerides in patients with MASLD, compared with less than 10% in healthy individuals [15,32].

A characteristic feature of MASLD is the persistence of DNL despite systemic insulin resistance, a phenomenon referred to as selective hepatic insulin resistance, in which insulin-dependent lipogenic signaling is preserved despite impaired suppression of gluconeogenesis. Consequently, chronic hyperinsulinemia continues to stimulate hepatic fatty acid synthesis, while high dietary fructose further enhances DNL by bypassing key glycolytic regulatory steps, increasing hepatic acetyl-CoA availability, and activating carbohydrate-responsive lipogenic pathways [15,16,23,32].

In addition to increasing hepatic triglyceride accumulation, excessive DNL alters intracellular lipid composition by promoting the formation of bioactive lipid intermediates, including diacylglycerols and ceramides, which impair insulin signaling, mitochondrial function, and inflammatory pathways, thereby linking metabolic overload to progressive liver injury [11,13,15]. These observations have established DNL as an important therapeutic target in MASLD, supporting the clinical development of inhibitors targeting ACC, FASN, and SCD1, as discussed later in this review [15,23].

#### 3.1.3. Dietary Lipid Uptake

Although dietary lipids contribute a smaller proportion of hepatic triglycerides than adipose tissue-derived free fatty acids or de novo lipogenesis, excessive dietary fat intake plays an important role in the initiation and progression of MASLD. In healthy individuals, dietary triglycerides are hydrolyzed in the intestinal lumen, absorbed by enterocytes, re-esterified, and incorporated into chylomicrons, which deliver triglycerides to peripheral tissues before chylomicron remnants are cleared by the liver. Under conditions of chronic overnutrition, increased intestinal lipid absorption and enhanced hepatic uptake of chylomicron remnants contribute to hepatic lipid accumulation and metabolic dysfunction (Figure 1) [15,16,33].

Intestinal lipid absorption is mediated by coordinated membrane transporters and intracellular lipid-handling proteins that regulate fatty acid uptake, cholesterol absorption, and chylomicron assembly. Alterations in the expression or activity of these pathways increase lipid delivery to the liver and contribute to the metabolic disturbances observed in MASLD [33,34].

Beyond the quantity of dietary fat, lipid quality substantially influences disease progression. Diets enriched in saturated fatty acids and cholesterol promote hepatic steatosis, lipotoxicity, oxidative stress, and inflammatory signaling to a greater extent than diets rich in mono- and polyunsaturated fatty acids. Excessive fructose intake further acts synergistically with dietary fat to accelerate hepatic lipid accumulation and enhance the generation of bioactive lipid species that promote metabolic dysfunction [12,15,23].

Recent evidence further highlights the importance of the gut–liver axis in regulating dietary lipid handling. Alterations in gut microbiota composition, intestinal permeability, bile acid metabolism, and chylomicron secretion may influence intestinal lipid absorption and hepatic lipid delivery, thereby contributing to MASLD progression. Microbiota-dependent changes in bile acid composition may additionally modulate intestinal and hepatic metabolic signaling, including FXR-dependent pathways, thereby linking the gut–liver axis to hepatic lipid and bile acid homeostasis. These mechanisms complement the hepatic FXR-mediated regulatory pathways discussed below [15,18,19,23].

#### 3.1.4. Impaired Fatty Acid Oxidation

Mitochondrial fatty acid β-oxidation is a central mechanism by which hepatocytes dispose of excess fatty acids and maintain lipid homeostasis. In MASLD, increased fatty acid delivery initially stimulates mitochondrial β-oxidation as an adaptive response to lipid overload. However, sustained metabolic stress progressively overwhelms this compensatory mechanism, particularly during the transition from simple steatosis to MASH, resulting in impaired oxidative lipid disposal [12,15,16,35].

Long-chain fatty acids are transported into mitochondria via the carnitine shuttle, in which carnitine palmitoyltransferase 1A (CPT1A) catalyzes the rate-limiting step. This process is tightly regulated by malonyl-CoA, linking de novo lipogenesis to fatty acid oxidation. In MASLD, enhanced lipogenesis increases malonyl-CoA availability, while insulin resistance, mitochondrial stress, and dysregulated AMPK–PPARα signaling further impair β-oxidation, redirecting fatty acids toward triglyceride storage and the synthesis of lipotoxic intermediates [15,18,19,32].

Impaired fatty acid oxidation promotes disease progression through multiple interconnected mechanisms. Reduced mitochondrial oxidative capacity leads to the accumulation of fatty acyl-CoAs, diacylglycerols, ceramides, and acylcarnitines, which disrupt insulin signaling and activate cellular stress pathways [11,13,23]. Simultaneously, incomplete β-oxidation and electron transport chain dysfunction increase reactive oxygen species production, resulting in oxidative injury, mitochondrial DNA damage, endoplasmic reticulum stress, and inflammatory activation [24,35,36]. Together, these processes amplify hepatocellular injury and promote progression toward steatohepatitis and fibrosis.

Human metabolic studies indicate that mitochondrial fatty acid oxidation is dynamically regulated across disease stages rather than uniformly impaired throughout MASLD. Although fatty acid oxidation may be preserved or even enhanced during early steatosis as a compensatory response to lipid overload, this adaptation becomes insufficient with disease progression, and patients with MASH exhibit impaired complete mitochondrial fatty acid oxidation, defective mitochondrial turnover, and increased reactive oxygen species production [35]. Thus, the contribution of fatty acid oxidation to MASLD is stage-dependent, shifting from an early adaptive response toward maladaptive mitochondrial dysfunction during progressive disease.

These observations underscore the importance of AMPK–PPARα signaling, mitochondrial biogenesis, mitophagy, and malonyl-CoA regulation in maintaining hepatic oxidative capacity and limiting lipid-induced cellular stress [17,18,19,35,36].

#### 3.1.5. Dysfunctional VLDL Secretion

Very-low-density lipoprotein (VLDL) secretion is the principal pathway by which the liver exports excess triglycerides and maintains intracellular lipid homeostasis. Under physiological conditions, triglycerides synthesized within hepatocytes are packaged together with apolipoprotein B100 (ApoB100), phospholipids, and cholesterol to form nascent VLDL particles in the endoplasmic reticulum. This tightly coordinated process depends on microsomal triglyceride transfer protein (MTP), which mediates ApoB100 lipidation and enables the assembly and secretion of mature VLDL particles [15,16,37].

VLDL secretion is also disease-stage dependent. During early MASLD, hepatic VLDL production may increase as a compensatory response to greater triglyceride availability; however, this adaptation has limited capacity and becomes insufficient as hepatic lipid overload and cellular dysfunction progress. In more advanced disease, impaired ApoB100 stability, MTP activity, and secretory function may further restrict lipid export, promoting intracellular triglyceride retention [15,23,37]. Thus, VLDL metabolism represents a dynamic adaptation rather than a uniformly defective pathway throughout MASLD progression.

Several mechanisms contribute to impaired VLDL secretion in advanced MASLD. Endoplasmic reticulum stress, oxidative stress, mitochondrial dysfunction, and chronic inflammation reduce ApoB100 stability and impair MTP activity, thereby limiting VLDL assembly and export [17,23,35]. In addition, insulin resistance disrupts ApoB100 degradation and lipoprotein metabolism, while excessive accumulation of saturated fatty acids and bioactive lipid intermediates further compromises the secretory machinery. As a result, triglycerides that cannot be efficiently exported accumulate within hepatocytes, promoting lipid droplet expansion and lipotoxic injury [11,15,16].

Importantly, increased VLDL secretion is not necessarily a beneficial adaptation, as enhanced hepatic export of triglyceride-rich lipoproteins contributes to the atherogenic dyslipidemia characteristic of MASLD, including elevated plasma triglycerides, increased concentrations of small dense low-density lipoprotein (LDL) particles, reduced high-density lipoprotein (HDL) cholesterol, and increased cardiovascular risk [2,7,24]. Thus, hepatic VLDL metabolism represents a complex metabolic trade-off between limiting intracellular lipid accumulation and promoting systemic lipid-mediated complications.

Recent lipidomic studies demonstrate that VLDL particles produced in MASLD exhibit distinct triglyceride, phospholipid, and sphingolipid profiles that reflect underlying disturbances in hepatic lipid metabolism and may serve as biomarkers of disease severity [25,29,38]. Collectively, dysfunctional VLDL secretion should be regarded not merely as a consequence of hepatic steatosis but as an integral component of hepatic lipid metabolic reprogramming linking intracellular lipid retention with systemic metabolic dysfunction.

### 3.2. Major Metabolic Regulators

The metabolic alterations described above are orchestrated by a highly integrated network of nutrient- and energy-sensing pathways that coordinate hepatic lipid synthesis, oxidation, storage, and export. Rather than functioning independently, these regulatory systems integrate hormonal signals, nutrient availability, and cellular energy status to maintain hepatic lipid homeostasis. In MASLD, chronic overnutrition, insulin resistance, and persistent metabolic stress disrupt this regulatory network, resulting in sustained lipogenesis, impaired fatty acid oxidation, defective lipid export, and accumulation of lipotoxic lipid species. Among the best-characterized regulators are sterol regulatory element-binding protein-1c (SREBP-1c), carbohydrate-responsive element-binding protein (ChREBP), AMP-activated protein kinase (AMPK), mechanistic target of rapamycin (mTOR), peroxisome proliferator-activated receptors (PPARs), and the farnesoid X receptor (FXR), which operate through extensive reciprocal crosstalk to form an integrated regulatory network coordinating hepatic metabolic adaptation (Figure 1) [15,16,18,19,23].

***Sterol regulatory element-binding protein-1c*** (SREBP-1c) is the principal transcription factor regulating hepatic de novo lipogenesis and a key driver of metabolic reprogramming in MASLD. SREBP-1c is activated primarily by insulin and nutrient excess and induces the expression of multiple lipogenic enzymes, including acetyl-CoA carboxylase (ACC), fatty acid synthase (FASN), stearoyl-CoA desaturase-1 (SCD1), and glycerol-3-phosphate acyltransferase (GPAT), thereby promoting hepatic fatty acid and triglyceride synthesis [15,16,23]. Together with ChREBP, AMPK, mTOR, PPARs, and FXR, SREBP-1c participates in an integrated nutrient-sensing network coordinating hepatic metabolic adaptation (Figure 1).

In MASLD, chronic hyperinsulinemia and nutrient overload result in persistent SREBP-1c activation despite systemic insulin resistance, leading to sustained lipogenesis and progressive hepatic steatosis [15,16,32]. SREBP-1c activity is further modulated through interactions with AMPK, mTORC1, LXR, and FXR, thereby integrating nutrient sensing with hepatic lipid metabolism [18,19]. Persistent SREBP-1c activation may not only promote triglyceride accumulation but also increase the generation of lipotoxic lipid intermediates that contribute to insulin resistance, inflammation, and fibrosis, making this pathway an important therapeutic target in MASLD [11,13,15,23].

***Carbohydrate-responsive element-binding protein*** (ChREBP) is a glucose-responsive transcription factor that links carbohydrate availability with hepatic de novo lipogenesis. Unlike SREBP-1c, which is primarily activated by insulin, ChREBP responds to intracellular glucose metabolites and induces the expression of glycolytic and lipogenic genes, including L-PK, ACC, FASN, and SCD1, thereby promoting the conversion of excess carbohydrates into fatty acids [15,16,23,39].

In MASLD, chronic consumption of carbohydrate-rich diets, particularly fructose, leads to sustained ChREBP activation and enhanced hepatic lipogenesis. Together, ChREBP and SREBP-1c coordinate the transcriptional program driving hepatic lipid accumulation and metabolic reprogramming [12,15,32]. Emerging evidence further indicates that ChREBP regulates hepatokine production and systemic glucose homeostasis, highlighting its broader role in metabolic regulation [39]. Accordingly, modulation of ChREBP activity has emerged as a potential therapeutic strategy for limiting excessive hepatic lipogenesis.

***AMP-activated protein kinase*** (AMPK) and the mechanistic target of rapamycin (mTOR) are key nutrient- and energy-sensing pathways that coordinate hepatic lipid metabolism. AMPK is activated by an increased intracellular AMP/ATP ratio during cellular energy deficiency, promoting catabolic processes, including mitochondrial fatty acid β-oxidation, while suppressing lipogenesis. Conversely, mechanistic target of rapamycin complex 1 (mTORC1) is activated by nutrient excess and insulin signaling, stimulating anabolic pathways such as lipid and protein synthesis [15,17,18,19].

In MASLD, reduced AMPK activity together with persistent mTORC1 activation disrupts hepatic metabolic homeostasis. AMPK directly phosphorylates and inhibits acetyl-CoA carboxylase (ACC), suppresses SREBP-1c-mediated lipogenesis, and promotes fatty acid oxidation, whereas mTORC1 enhances lipogenic gene expression and inhibits autophagy. The resulting imbalance favors triglyceride accumulation, mitochondrial dysfunction, and progressive lipotoxicity [15,17,18,19].

Beyond regulating lipid metabolism, the AMPK–mTOR axis influences mitochondrial quality control, oxidative stress, autophagy, and inflammatory signaling, thereby linking nutrient sensing to MASLD progression [17,18,19,23,40].

***Peroxisome proliferator-activated receptors*** (PPARs) are ligand-activated nuclear receptors that regulate hepatic lipid metabolism, energy homeostasis, and inflammation. Of the three isoforms, PPARα is predominantly expressed in the liver and promotes mitochondrial and peroxisomal fatty acid β-oxidation, whereas PPARγ regulates insulin sensitivity and lipid storage, and PPARδ contributes to metabolic flexibility by promoting fatty acid utilization, mitochondrial oxidative metabolism, and improved insulin sensitivity in multiple metabolic tissues [18,19].

In MASLD, reduced PPARα activity impairs fatty acid oxidation and promotes triglyceride accumulation. Conversely, PPARα activation enhances fatty acid transport and β-oxidation, whereas PPARγ primarily improves insulin sensitivity and promotes lipid storage in adipose tissue, thereby indirectly reducing ectopic hepatic lipid accumulation. PPARδ also contributes to hepatic lipid utilization and metabolic flexibility, although its role in MASLD remains less well defined [15,18,19].

Collectively, PPAR signaling integrates lipid metabolism with inflammatory and fibrotic pathways. Several dual- and pan-PPAR agonists have demonstrated metabolic and histological effects in clinical trials and are discussed in Section 10 [15,18,19,41].

***Farnesoid X receptor*** (FXR) is a bile acid-activated nuclear receptor that plays a central role in maintaining hepatic lipid, glucose, and bile acid homeostasis. Upon activation by bile acids, FXR suppresses hepatic de novo lipogenesis, promotes fatty acid β-oxidation, and limits bile acid synthesis by inducing the small heterodimer partner (SHP), which inhibits cholesterol 7α-hydroxylase (CYP7A1), the rate-limiting enzyme in bile acid biosynthesis [18,19,42]. FXR activation also induces fibroblast growth factor 19 (FGF19) signaling, thereby further suppressing hepatic bile acid synthesis and contributing to systemic regulation of glucose and lipid metabolism.

Altered FXR signaling has been implicated in hepatic lipid accumulation, insulin resistance, inflammation, and progressive liver injury in MASLD; however, its effects are context dependent and influenced by bile acid composition, tissue-specific signaling, and disease stage.

Reduced FXR activity is associated with increased SREBP-1c-mediated lipogenesis, altered bile acid composition, and disruption of the gut–liver axis, thereby amplifying metabolic dysfunction [15,18,19]. In addition to its metabolic functions, FXR modulates inflammatory and fibrogenic pathways through interactions with NF-κB and other nuclear receptors, linking bile acid signaling to disease progression.

FXR agonists have demonstrated metabolic and histological effects in preclinical and clinical studies, although their long-term efficacy, tolerability, and safety remain important limitations [18,19,42].

Collectively, these nutrient- and energy-sensing pathways function as an integrated regulatory network linking metabolic adaptation with lipotoxicity, inflammation, and fibrosis, thereby driving disease progression and representing major targets for mechanism-based therapeutic intervention in MASLD.

## 4. Lipotoxic Lipid Species in MASLD

Hepatic lipid accumulation in MASLD is characterized by both quantitative and qualitative alterations in hepatic lipid composition. Several bioactive lipid species exert lipotoxic effects that drive hepatocellular injury and disease progression. Saturated fatty acids, ceramides, sphingolipids, diacylglycerols, oxidized lipids, and free cholesterol disrupt mitochondrial function, induce endoplasmic reticulum stress, activate inflammatory signaling pathways, and promote fibrogenesis. The abundance, intracellular distribution, and dynamic interactions among these bioactive lipid species are now recognized as key determinants of disease severity, highlighting lipotoxicity as an integrated signaling network linking metabolic dysfunction to progressive liver injury. (Table 1) [11,12,13,14,15,16,20,21,22,23,24,25].

### 4.1. Saturated Fatty Acids

Although triglycerides constitute the predominant lipid stored in steatotic hepatocytes, their accumulation is not uniformly cytotoxic. Under conditions in which lipid-droplet storage capacity is preserved, triglyceride synthesis may serve as an adaptive buffering mechanism by sequestering excess fatty acids and limiting their conversion into more bioactive lipotoxic intermediates. In contrast, saturated fatty acids (SFAs), particularly palmitic acid (C16:0), are key mediators of hepatocellular injury and play a pivotal role in the progression from simple steatosis to MASH [11,12,13,15,43]. Rather than serving solely as metabolic substrates, SFAs act as bioactive molecules that disrupt multiple pathways essential for hepatic homeostasis (Table 1).

The hepatotoxicity of SFAs reflects their limited capacity for safe esterification and storage compared with monounsaturated fatty acids. Insufficient stearoyl-CoA desaturase-1 (SCD1) activity further limits the conversion of saturated fatty acids into monounsaturated fatty acids, thereby reducing their safe incorporation into triglycerides and increasing lipotoxic potential. When the buffering capacity of lipid droplets is exceeded, excess palmitate is redirected toward the synthesis of toxic lipid intermediates or incorporated into cellular membranes, leading to membrane rigidity, organelle dysfunction, and activation of stress signaling pathways [11,13,43]. In contrast, monounsaturated fatty acids such as oleate are more efficiently incorporated into triglycerides, thereby attenuating lipotoxic stress and protecting hepatocytes from SFA-induced injury.

Excessive SFA accumulation induces endoplasmic reticulum stress, mitochondrial dysfunction, oxidative stress, and inflammatory activation through multiple interconnected mechanisms. Palmitate promotes reactive oxygen species production, impairs mitochondrial respiration, disrupts calcium homeostasis, and activates stress-responsive pathways, including c-Jun N-terminal kinase (JNK), nuclear factor-κB (NF-κB), and the NLRP3 inflammasome [11,12,13,21,23]. These responses drive hepatocyte apoptosis, inflammatory cell recruitment, and hepatic stellate cell activation, thereby promoting fibrosis.

In addition to their direct cytotoxic effects, SFAs serve as precursors for ceramides, diacylglycerols, and other bioactive lipid species that further amplify lipotoxic signaling. These secondary lipid mediators disrupt insulin signaling, impair mitochondrial function, and sustain inflammatory responses, placing SFAs among the principal initiators of the lipotoxic cascade underlying MASLD progression and providing the mechanistic basis for the following sections on specific bioactive lipid classes [13,20,21,22,23].

### 4.2. Ceramides

Ceramides are bioactive sphingolipids that have emerged as central mediators linking lipid overload to hepatocellular dysfunction in MASLD. Unlike triglycerides, which may serve an adaptive buffering role by sequestering excess fatty acids under conditions of preserved lipid-storage capacity, ceramides regulate multiple intracellular signaling pathways involved in insulin resistance, oxidative stress, inflammation, apoptosis, and fibrosis. Hepatic ceramide accumulation results from enhanced de novo synthesis driven by excessive saturated fatty acid availability, sphingomyelin hydrolysis, and activation of the sphingolipid salvage pathway, all of which are upregulated under conditions of chronic metabolic stress [11,13,15,23,44].

The de novo ceramide biosynthetic pathway is initiated by the condensation of serine and palmitoyl-CoA via serine palmitoyltransferase (SPT), followed by sequential reactions catalyzed by ceramide synthases (CerS1–6) and dihydroceramide desaturase (DES1). Individual ceramide species exert distinct biological effects depending on their acyl-chain length and tissue distribution. In particular, C16:0 ceramide, predominantly generated by CerS6, has been strongly associated with hepatic insulin resistance, mitochondrial dysfunction, and lipotoxicity, whereas very-long-chain ceramides may exert partially protective effects, highlighting the functional heterogeneity of ceramide metabolism [13,44].

Ceramides function as key signaling molecules that promote hepatocellular injury. Increased intracellular ceramide levels inhibit insulin signaling through suppression of Akt phosphorylation and activation of protein phosphatase 2A (PP2A), thereby promoting hepatic insulin resistance. They also impair mitochondrial respiration, enhance reactive oxygen species production, induce endoplasmic reticulum stress, and activate stress-responsive signaling pathways that contribute to hepatocyte injury and inflammation [11,13,21,23].

Beyond these metabolic effects, ceramides amplify inflammatory and fibrogenic signaling. Elevated hepatic ceramide concentrations have been associated with activation of c-Jun N-terminal kinase (JNK), nuclear factor-κB (NF-κB), and the NLRP3 inflammasome, thereby linking lipid metabolic dysregulation to chronic hepatic inflammation and fibrosis [11,21,23,44]. These signaling pathways are discussed in greater detail in the following sections of this review.

Recent targeted and untargeted lipidomic studies demonstrate disease-associated alterations in circulating and hepatic ceramide profiles in MASLD [25,26]. Importantly, the relative abundance and composition of individual ceramide species may provide greater biological and potentially diagnostic information than total ceramide content alone. This distinction is mechanistically plausible because ceramide species with different acyl-chain lengths exert distinct and, in some contexts, divergent metabolic effects [13,14,21]. Thus, changes in total ceramide abundance may not adequately reflect the balance among ceramide species with different biological properties. Species-resolved ceramide profiling may therefore better capture the molecular heterogeneity of MASLD and its relationship with metabolic dysfunction and histological disease severity [21,25,26,45]. Nevertheless, individual ceramide species or ceramide ratios have not yet been established as clinically validated biomarkers. Prospective studies integrating hepatic and circulating ceramide profiles are required to determine whether species-specific signatures provide clinically meaningful information beyond total ceramide concentrations and established non-invasive markers [25,26,45].

### 4.3. Sphingolipids

Beyond ceramides, the sphingolipid family comprises a diverse group of bioactive lipids, including sphingomyelins, sphingosine, sphingosine-1-phosphate (S1P), and glycosphingolipids, which regulate membrane organization, cell survival, inflammation, and immune responses. Rather than acting as independent molecules, these metabolites constitute an interconnected metabolic network in which alterations in one sphingolipid species influence the cellular balance of others [11,20,44,45]. Increasing evidence indicates that dysregulation of sphingolipid metabolism is a hallmark of MASLD progression and contributes to hepatocellular injury beyond the effects of ceramide accumulation alone.

Among these metabolites, S1P has attracted particular attention because it frequently exerts biological effects opposite to those of ceramides. Whereas ceramides generally promote apoptosis, oxidative stress, and insulin resistance, the biological effects of S1P are highly receptor-dependent and vary according to the relative expression of five G protein-coupled S1P receptors (S1PR1–S1PR5) in different hepatic cell populations. However, persistent activation of the sphingosine kinase (SphK)/S1P signaling axis under chronic metabolic stress can also promote inflammatory cell recruitment, hepatic stellate cell activation, and fibrogenesis, underscoring the context-dependent nature of S1P signaling [20,44,45].

This receptor- and cell-specific heterogeneity represents an important challenge for therapeutic targeting of the S1P/S1PR axis in MASLD. Because individual S1P receptor subtypes may mediate distinct or even opposing effects across hepatic cell populations, non-selective modulation of S1P signaling could result in counteracting biological responses. Receptor-subtype- and cell-specific approaches may therefore offer greater therapeutic precision than global modulation of the S1P/S1PR axis. However, current evidence remains largely preclinical and insufficient to identify a single S1P receptor subtype as a validated or clearly superior therapeutic target in MASLD. Further cell-specific, disease-stage-specific, and translational studies are required before S1PR-directed strategies can be reliably implemented in clinical practice [20,45].

Altered sphingomyelin metabolism further contributes to MASLD pathogenesis. Enhanced sphingomyelin hydrolysis by acid and neutral sphingomyelinases increases ceramide generation, thereby amplifying lipotoxic signaling and inflammatory responses. In parallel, lipidomic studies have identified significant alterations in circulating and hepatic sphingomyelin species that correlate with steatohepatitis severity and fibrosis stage, supporting their potential utility as diagnostic biomarkers [25,26,29,45].

Collectively, sphingolipid metabolism represents a dynamic signaling network linking lipid homeostasis with inflammation, fibrosis, and cellular stress responses in MASLD [44,45].

### 4.4. Diacylglycerols

Diacylglycerols (DAGs) are bioactive lipid intermediates that accumulate in the liver during metabolic overload and contribute to hepatic insulin resistance. In addition to serving as precursors for triglyceride and phospholipid synthesis, DAGs act as intracellular second messengers regulating multiple signaling pathways involved in glucose and lipid metabolism [3,11,15,23].

In MASLD, excessive free fatty acid influx and enhanced de novo lipogenesis promote hepatic DAG accumulation, particularly within the plasma membrane and endoplasmic reticulum, where DAGs activate novel protein kinase C (PKC) isoforms. Among these, PKCε has emerged as a critical mediator of hepatic insulin resistance by inhibiting insulin receptor kinase activity and attenuating downstream Akt signaling, thereby impairing hepatic glucose metabolism while promoting further lipid accumulation [3,15,23,46].

Importantly, the metabolic effects of DAGs depend not only on total abundance but also on molecular species and subcellular localization. Membrane-associated DAGs, particularly within compartments accessible to PKCε, appear to have greater signaling potential than DAGs sequestered within lipid droplets, which may be comparatively metabolically inert. Accordingly, total hepatic DAG content does not necessarily reflect pathogenic signaling activity. Experimental and human lipidomic studies suggest that specific DAG species and their compartmental distribution are more closely associated with hepatic insulin resistance than total DAG abundance; however, their relationship with histological disease severity and progression remains incompletely defined [25,29,46].

Beyond their direct effects on insulin signaling, DAGs cooperate with saturated fatty acids and ceramides to amplify lipotoxic stress and inflammatory responses. This interplay establishes a mechanistic link between hepatic lipid accumulation and dysregulated intracellular signaling, providing the molecular basis for the PKCε-dependent signaling pathways discussed in the following section [11,13,21,23].

### 4.5. Oxidized Lipids

Oxidative modification of lipids generates a heterogeneous group of bioactive molecules that substantially contribute to MASLD progression. Oxidized phospholipids (OxPLs), oxidized cholesterol derivatives (oxysterols), and oxidized lipoproteins are formed under conditions of increased reactive oxygen species production and lipid peroxidation. Rather than representing passive by-products of oxidative stress, these molecules function as potent signaling mediators that regulate inflammation, innate immunity, and cellular stress responses [11,24,47]. Reactive lipid peroxidation products such as 4-hydroxynonenal (4-HNE) and malondialdehyde (MDA) further amplify oxidative injury by modifying proteins, nucleic acids, and membrane phospholipids.

Oxidized lipids contain oxidation-specific epitopes (OSEs), which are recognized as damage-associated molecular patterns (DAMPs) by pattern-recognition receptors expressed on Kupffer cells, macrophages, dendritic cells, and other immune cells. Activation of these receptors stimulates pro-inflammatory signaling pathways, promotes cytokine production, and enhances immune cell recruitment, thereby linking lipid peroxidation to chronic hepatic inflammation [20,24,47]. In addition, oxidized phospholipids alter membrane integrity and mitochondrial function, further amplifying oxidative injury and hepatocellular dysfunction.

Recent lipidomic studies have demonstrated that specific oxidized lipid species correlate with steatohepatitis severity, fibrosis progression, and cardiovascular risk in patients with MASLD, supporting their potential utility as diagnostic and prognostic biomarkers [24,25,26,27,28,29]. Moreover, increasing evidence indicates that oxidized lipids actively participate in intercellular communication and immune modulation, highlighting their emerging role as therapeutic targets. Together, these mechanisms link hepatic lipid metabolic reprogramming with oxidative injury and the inflammatory signaling pathways discussed in the following chapter.

### 4.6. Cholesterol Toxicity

Although cholesterol is an essential structural component of cellular membranes and a precursor of steroid hormones and bile acids, excessive accumulation of free cholesterol has emerged as an important contributor to lipotoxicity in MASLD. Unlike cholesteryl esters, which are relatively inert storage forms, unesterified cholesterol readily incorporates into cellular and organelle membranes, altering membrane fluidity and disrupting intracellular homeostasis [15,23,24].

Under physiological conditions, hepatocytes maintain cholesterol homeostasis through coordinated regulation of cholesterol synthesis, uptake, esterification, biliary secretion, and conversion to bile acids. Disrupted intracellular cholesterol trafficking, particularly impaired lysosomal cholesterol export, further contributes to free cholesterol accumulation within vulnerable intracellular compartments. Elevated free cholesterol induces endoplasmic reticulum stress, mitochondrial dysfunction, oxidative injury, and lysosomal membrane destabilization, thereby increasing hepatocyte susceptibility to apoptosis and inflammatory activation [15,24,48].

Free cholesterol also plays a critical role in the activation of hepatic macrophages and stellate cells. Cholesterol crystal accumulation within Kupffer cells stimulates NLRP3 inflammasome activation and pro-inflammatory cytokine release, whereas cholesterol loading of hepatic stellate cells enhances their activation and extracellular matrix production, thereby promoting fibrogenesis [21,24,48]. In parallel, altered cholesterol metabolism influences membrane lipid raft organization, modulating receptor-mediated signaling pathways involved in inflammation and immune responses.

Recent lipidomic and clinical studies indicate that hepatic free cholesterol correlates more closely with steatohepatitis severity and fibrosis progression than total hepatic lipid content [15,23,24,48]. These findings support free cholesterol as an active pathogenic mediator linking disrupted cholesterol homeostasis to lipotoxicity and MASLD progression [15,23,24,48].

Collectively, these bioactive lipid species form an interconnected lipotoxic signaling network in which metabolic, inflammatory, oxidative, and fibrogenic pathways continuously reinforce one another. Understanding this molecular interplay provides the conceptual basis for the signaling networks and organelle dysfunction discussed in the following sections of this review.

## 5. Lipid Signaling Networks Driving Disease Progression

**Figure 2:**
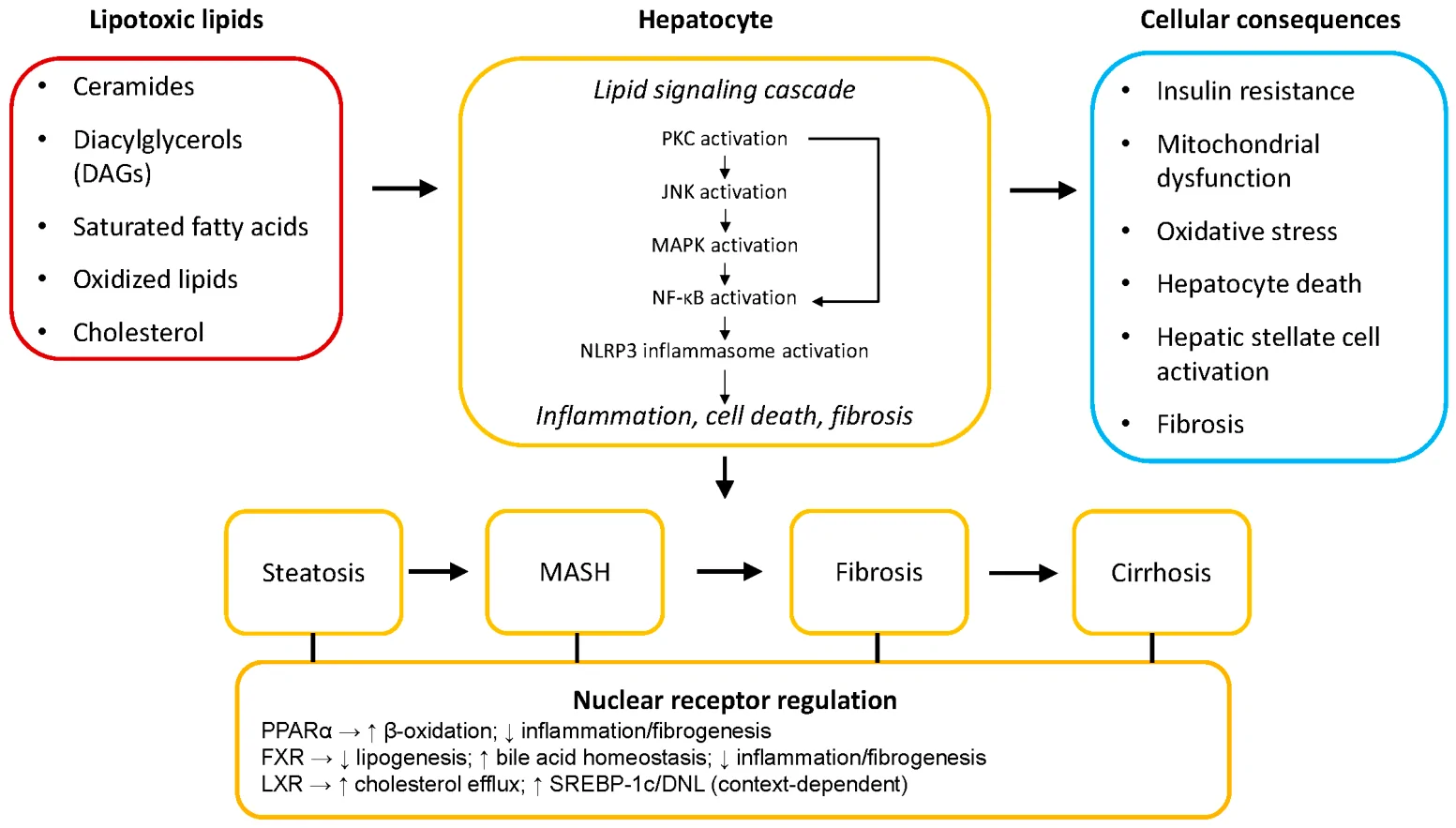
Lipid signaling networks driving MASLD progression. Bioactive lipid species activate interconnected intracellular signaling pathways, including PKC, MAPK, JNK, NF-κB, and the NLRP3 inflammasome, thereby promoting insulin resistance, oxidative stress, inflammation, hepatocyte injury, and hepatic stellate cell activation. Crosstalk among these signaling cascades establishes interconnected stress and inflammatory networks that may contribute to progression toward MASH and fibrosis in susceptible patients, with substantial interindividual and disease-stage-dependent variability.

Hepatic lipid accumulation in MASLD not only reflects metabolic imbalance but also reshapes intracellular signaling networks that regulate cell survival, inflammation, metabolism, and tissue remodeling. Bioactive lipid species, including saturated fatty acids, ceramides, diacylglycerols, oxidized lipids, and free cholesterol, function as signaling molecules that modulate stress-responsive kinases, inflammatory pathways, and nuclear receptors. These lipid-mediated signaling cascades integrate metabolic stress with immune activation, hepatocellular injury, and fibrogenesis, thereby driving the transition from simple steatosis to MASH. Rather than operating independently, these pathways form an interconnected signaling network characterized by extensive crosstalk and positive feedback mechanisms that amplify disease progression while simultaneously providing multiple opportunities for mechanism-based therapeutic intervention (Figure 2) [11,12,13,14,15,16,17,18,19,20,21,22,23,24].

### 5.1. Protein Kinase C Signaling

Protein kinase C (PKC) signaling represents one of the earliest molecular events linking hepatic lipid accumulation to metabolic dysfunction in MASLD. Among PKC family members, the novel isoform PKCε is the principal mediator of diacylglycerol (DAG)-induced hepatic insulin resistance, whereas PKCδ and several conventional PKC isoforms contribute to oxidative stress, inflammatory signaling, and hepatocyte injury [3,15,23,46].

Increased hepatic DAG accumulation promotes the translocation of PKCε from the cytosol to cellular membranes, where it inhibits insulin receptor kinase activity and attenuates downstream insulin signaling. This reduces Akt phosphorylation and activation, disrupts hepatic glucose metabolism, and further enhances lipogenesis, thereby establishing a vicious cycle between lipid accumulation and insulin resistance [3,15,46]. Beyond DAGs, ceramides further amplify PKC-dependent signaling through interactions with membrane lipid microdomains and stress-associated kinase networks, highlighting the coordinated actions of multiple lipotoxic lipid species [13,21,44].

In addition to its metabolic effects, PKC activation promotes reactive oxygen species generation, mitochondrial dysfunction, and activation of pro-inflammatory pathways, including NF-κB and JNK signaling. While PKCε predominantly mediates metabolic dysfunction and hepatic insulin resistance, PKCδ has been more closely associated with oxidative stress, mitochondrial injury, and hepatocyte apoptosis, underscoring the isoform-specific functions of PKC signaling in MASLD [11,15,21,23].

### 5.2. JNK Signaling

c-Jun N-terminal kinase (JNK) is a stress-activated serine/threonine kinase and a central mediator linking lipotoxicity to hepatocellular injury in MASLD. Hepatic JNK activation is triggered by multiple lipotoxic stimuli, including saturated fatty acids, ceramides, oxidized lipids, reactive oxygen species, and endoplasmic reticulum stress, placing this pathway at the intersection of metabolic and inflammatory signaling [11,13,21,23].

Upon activation, JNK phosphorylates insulin receptor substrate-1 (IRS-1) at inhibitory serine residues, thereby impairing insulin signaling and aggravating hepatic insulin resistance. In parallel, JNK regulates the transcriptional activity of activator protein-1 (AP-1), promoting the expression of pro-inflammatory cytokines, chemokines, and stress-responsive genes that contribute to hepatocyte dysfunction and immune cell recruitment [15,21,23]. Persistent JNK activation further exacerbates mitochondrial dysfunction and oxidative stress, establishing a positive feedback loop that further amplifies lipotoxic injury.

JNK signaling additionally promotes hepatocyte apoptosis by modulating Bcl-2 family proteins and activating mitochondrial cell death pathways. Experimental studies further indicate that JNK1 appears to play a predominant role in hepatic steatosis and insulin resistance, whereas JNK2 may contribute more directly to hepatocyte injury and apoptosis, although considerable overlap exists between these isoforms [21,23]. Crosstalk between JNK and NF-κB, MAPK, and NLRP3 inflammasome signaling further integrates lipid-induced stress responses with chronic inflammation and fibrogenesis [11,21,24]. Consequently, sustained JNK activation represents an important molecular link between hepatic lipid metabolic dysregulation, inflammatory signaling, and disease progression.

### 5.3. MAPK Signaling

Mitogen-activated protein kinase (MAPK) signaling is a highly conserved regulatory network that integrates extracellular stimuli with intracellular responses governing cell proliferation, differentiation, inflammation, and survival. In MASLD, excessive lipid accumulation, oxidative stress, inflammatory cytokines, and mitochondrial dysfunction activate multiple MAPK pathways, particularly extracellular signal-regulated kinase (ERK1/2) and p38 MAPK, thereby promoting hepatocellular injury and disease progression [11,15,23].

ERK1/2 signaling is primarily activated by growth factors and metabolic stimuli and regulates hepatocyte proliferation, lipid metabolism, and cell survival [21,24]. Under chronic metabolic stress, sustained ERK1/2 activation enhances lipogenic gene expression and inflammatory responses, contributing to hepatic lipid accumulation. In contrast, p38 MAPK is predominantly activated by oxidative stress and pro-inflammatory cytokines and regulates cytokine production, apoptosis, and fibrogenic signaling [21,24]. Together, sustained ERK1/2 and p38 activation amplifies the cellular response to lipotoxic stress and promotes the transition from simple steatosis to steatohepatitis.

MAPK signaling exhibits extensive crosstalk with other lipid-responsive pathways, including PKC, JNK, NF-κB, and the NLRP3 inflammasome. Through these interactions, MAPKs integrate metabolic stress with inflammatory and profibrotic signaling, contributing to hepatocyte dysfunction, immune cell recruitment, and hepatic stellate cell activation. Consequently, modulation of MAPK signaling may emerge as a potential therapeutic strategy for attenuating lipid-induced liver injury and slowing MASLD progression [11,21,23,24].

### 5.4. NF-κB Signaling

Nuclear factor-κB (NF-κB) is a master transcriptional regulator of inflammation and innate immune responses that plays a central role in MASLD progression. Under physiological conditions, NF-κB remains inactive in the cytoplasm through its association with inhibitor of κB (IκB) proteins. However, chronic metabolic stress, lipotoxic lipid species, oxidative stress, and pro-inflammatory cytokines promote IκB phosphorylation and degradation, allowing NF-κB to translocate to the nucleus and initiate transcription of numerous inflammatory genes [11,15,23].

Multiple bioactive lipid species contribute to NF-κB activation in MASLD. Saturated fatty acids can enhance Toll-like receptor 4 (TLR4)—MyD88—associated inflammatory signaling, particularly under conditions of metabolic stress, whereas ceramides, oxidized phospholipids, and free cholesterol amplify inflammatory responses through stress-associated kinase pathways and pattern-recognition receptors [20,21,24]. These converging signals induce the expression of tumor necrosis factor-α (TNF-α), interleukin-6 (IL-6), monocyte chemoattractant protein-1 (MCP-1), and other pro-inflammatory mediators that promote immune cell recruitment and hepatocellular injury [11,20,21,24].

Beyond regulating inflammatory gene expression, NF-κB integrates lipid metabolic dysregulation with oxidative stress, endoplasmic reticulum stress, and mitochondrial dysfunction. Extensive crosstalk with PKC, JNK, MAPK, and nuclear receptor signaling further amplifies chronic inflammatory responses and promotes hepatic stellate cell activation and fibrogenesis. In addition, NF-κB provides the priming signal required for subsequent activation of the NLRP3 inflammasome, thereby linking lipid-induced inflammatory signaling to innate immune activation [21,23,24].

Persistent NF-κB activation has consistently been associated with increased disease severity, progression to steatohepatitis, and liver fibrosis. Accordingly, therapeutic strategies targeting upstream lipid-mediated activation or selectively modulating NF-κB signaling have attracted considerable interest as potential approaches for limiting chronic inflammation and slowing MASLD progression [15,18,23].

### 5.5. NLRP3 Inflammasome Activation

The nucleotide-binding oligomerization domain-like receptor family pyrin domain-containing 3 (NLRP3) inflammasome is a key component of the innate immune system linking lipid-induced metabolic stress to chronic hepatic inflammation in MASLD. It is a multiprotein complex composed of the NLRP3 sensor, the adaptor protein apoptosis-associated speck-like protein containing a caspase recruitment domain (ASC), and pro-caspase-1. Upon activation, caspase-1 is cleaved into its active form, promoting the maturation and secretion of the pro-inflammatory cytokines interleukin-1β (IL-1β) and interleukin-18 (IL-18), while simultaneously inducing pyroptotic cell death [11,21,23].

Lipotoxic lipid species provide both the priming and activation signals required for inflammasome assembly. Saturated fatty acids, ceramides, oxidized phospholipids, and free cholesterol crystals stimulate pattern-recognition receptors and activate NF-κB, inducing the transcription of NLRP3 and pro-IL-1β [11,21]. Subsequent mitochondrial dysfunction, reactive oxygen species generation, lysosomal destabilization, potassium efflux, and mitochondrial DNA release provide the second signal, triggering inflammasome assembly and caspase-1 activation [11,21,24,48].

Beyond hepatocytes, NLRP3 inflammasome activation occurs in Kupffer cells, infiltrating macrophages, hepatic stellate cells, and liver sinusoidal endothelial cells, amplifying inflammatory communication within the hepatic microenvironment. Persistent inflammasome activation enhances cytokine production, promotes immune cell recruitment, accelerates hepatocyte injury, and stimulates hepatic stellate cell activation, thereby driving fibrosis progression [15,23,24].

Emerging evidence identifies NLRP3 signaling as a critical convergence point linking lipotoxicity, metabolic dysfunction, and innate immunity. Accordingly, pharmacological inhibition of inflammasome activation, caspase-1 activity, or IL-1β signaling may provide a potential approach to limiting chronic inflammation and disease progression [21,23,24]. However, the contribution of NLRP3 signaling varies across hepatic cell populations and experimental models, and its relative importance may differ according to metabolic context and disease stage.

### 5.6. Nuclear Receptor Signaling

Nuclear receptors function as lipid-sensing transcription factors that integrate metabolic and inflammatory signaling in the liver (Figure 2). By responding to fatty acids, oxysterols, bile acids, and other lipid-derived ligands, they regulate genes involved in lipid synthesis, fatty acid oxidation, cholesterol homeostasis, and immune responses. Rather than acting independently, peroxisome proliferator-activated receptors (PPARs), farnesoid X receptor (FXR), liver X receptors (LXRs), and retinoid X receptors (RXRs) form an interconnected regulatory network that coordinates hepatic adaptation to metabolic stress [15,18,19,23].

PPARα is the principal regulator of hepatic fatty acid β-oxidation, whereas PPARγ primarily regulates adipogenesis, insulin sensitivity, and lipid storage, thereby indirectly limiting ectopic hepatic lipid accumulation. FXR suppresses hepatic lipogenesis while maintaining bile acid and cholesterol homeostasis, whereas LXR functions as a sterol sensor that promotes cholesterol efflux but also enhances SREBP-1c-dependent lipogenesis under conditions of nutrient excess [18,19].

RXRs serve as obligate heterodimerization partners for several nuclear receptors, including PPARs, FXR, and LXRs, thereby coordinating transcriptional responses to multiple lipid-derived signals. Crosstalk among these receptor systems enables fine regulation of lipid metabolism but also contributes to persistent lipogenesis, impaired fatty acid oxidation, altered cholesterol metabolism, chronic inflammation, and progressive fibrosis in MASLD [15,18,19,23].

Beyond metabolic regulation, nuclear receptors directly modulate inflammatory signaling through reciprocal interactions with NF-κB, AP-1, and other transcriptional regulators, linking lipid sensing to innate immunity and tissue remodeling. Given their central role in hepatic metabolic homeostasis, several nuclear receptors may become as attractive pharmacological targets [18,19,23].

Collectively, lipid-responsive signaling pathways and nuclear receptor networks integrate metabolic adaptation with inflammatory, oxidative, and fibrogenic responses, thereby establishing the molecular framework that links hepatic lipid metabolic reprogramming to progressive organelle dysfunction discussed in the following chapter.

## 6. Organelle Dysfunction and Cellular Stress Responses

**Figure 3:**
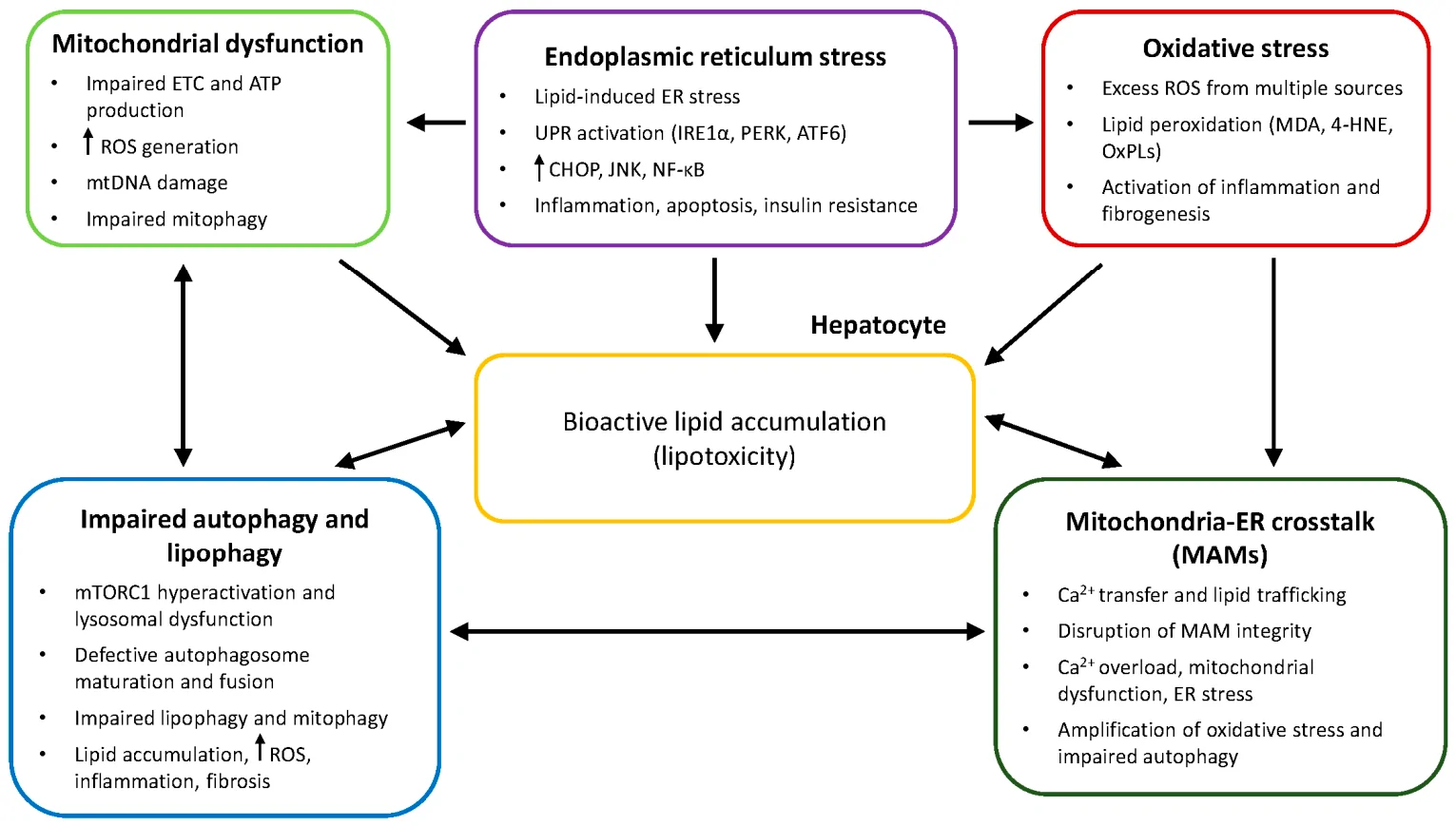
Organelle dysfunction and interconnected stress responses in MASLD. Chronic lipotoxicity induces coordinated dysfunction of mitochondria, the endoplasmic reticulum, lysosomes, and mitochondria-associated membranes (MAMs), resulting in impaired autophagy, oxidative stress, calcium dysregulation, and activation of the unfolded protein response. Extensive crosstalk among these organelles amplifies hepatocellular injury, inflammation, and fibrogenesis, thereby promoting progressive disease severity in MASLD.

Persistent lipid overload in MASLD disrupts the structure and function of major cellular organelles. Organelle dysfunction is not merely a consequence of lipotoxicity but also actively amplifies intracellular signaling pathways that drive disease progression. Lipotoxic lipid species, including saturated fatty acids, ceramides, oxidized lipids, and free cholesterol, impair mitochondrial function, induce endoplasmic reticulum (ER) stress, enhance oxidative stress, and compromise cellular quality control mechanisms. These interconnected processes amplify inflammation, hepatocellular injury, and fibrogenesis, thereby contributing to progression from simple steatosis to MASH in susceptible patients. The following sections summarize the major organelle-specific stress responses and their contributions to MASLD pathogenesis (Figure 3) [11,15,17,21,23,24,49].

### 6.1. Mitochondrial Dysfunction

Mitochondria are central regulators of hepatic energy metabolism, coordinating fatty acid β-oxidation, oxidative phosphorylation, ATP production, and reactive oxygen species (ROS) homeostasis. During the early stages of MASLD, mitochondria undergo adaptive remodeling to accommodate increased lipid availability by enhancing fatty acid oxidation and respiratory activity. However, persistent lipid overload eventually exceeds mitochondrial metabolic capacity, leading to impaired oxidative phosphorylation, defective ATP production, excessive ROS generation, and mitochondrial DNA damage, thereby promoting hepatocellular dysfunction (Figure 3) [15,17,23,24,49].

Lipotoxic lipid species directly disrupt mitochondrial integrity by altering membrane composition, impairing electron transport chain activity, and promoting calcium overload. These alterations reduce mitochondrial respiratory efficiency while increasing electron leakage and oxidative stress, establishing a self-amplifying cycle between mitochondrial dysfunction and chronic lipotoxicity [11,13,21,24,44,49].

Dysregulated mitochondrial dynamics further contribute to disease progression. Excessive mitochondrial fission, impaired fusion, defective mitophagy, and reduced mitochondrial biogenesis promote the accumulation of dysfunctional mitochondria, sustaining oxidative stress and inflammatory signaling. Moreover, damaged mitochondria release mitochondrial DNA and other damage-associated molecular patterns (DAMPs), which activate innate immune pathways, including the NLRP3 inflammasome, further amplifying hepatocellular injury and fibrosis [21,23,24,49,50].

Collectively, mitochondrial dysfunction represents a central event linking lipid metabolic dysregulation with oxidative stress, inflammation, and hepatocyte death. Accordingly, therapeutic strategies aimed at restoring mitochondrial bioenergetics, improving mitochondrial quality control, stimulating mitochondrial biogenesis, and enhancing mitophagy may prove to be promising approaches for slowing MASLD progression [23,24,49,50].

### 6.2. Endoplasmic Reticulum Stress

The endoplasmic reticulum (ER) is essential for protein folding, lipid biosynthesis, calcium homeostasis, and cellular metabolic regulation. Under physiological conditions, the adaptive unfolded protein response (UPR) maintains ER homeostasis by restoring protein-folding capacity. In MASLD, however, chronic lipid overload overwhelms these adaptive mechanisms, resulting in persistent ER stress and activation of maladaptive UPR pathways that contribute to hepatocellular injury [11,15,17,21,23,24,49,50,51].

Lipotoxic lipid species disrupt ER membrane integrity, alter calcium homeostasis, and promote protein misfolding. These alterations activate the three principal UPR sensors—inositol-requiring enzyme 1α (IRE1α), protein kinase RNA-like ER kinase (PERK), and activating transcription factor 6 (ATF6). Although transient activation of these pathways is initially protective, sustained ER stress induces C/EBP homologous protein (CHOP), c-Jun N-terminal kinase (JNK), and nuclear factor-κB (NF-κB) signaling, promoting inflammation, apoptosis, and metabolic dysfunction [11,21,23,24,51].

ER stress is closely interconnected with mitochondrial dysfunction through mitochondria-associated ER membranes (MAMs), where disturbances in calcium transfer and reactive oxygen species production amplify organelle dysfunction and lipotoxic injury. Prolonged UPR activation also impairs insulin signaling, enhances hepatic lipogenesis, and promotes inflammatory cytokine production, establishing a feed-forward cycle linking metabolic stress to progressive liver damage [15,17,23,24,49,50].

Collectively, chronic ER stress represents a central mechanism by which lipotoxic lipids couple metabolic dysregulation with inflammatory signaling and hepatocyte death. Accordingly, therapeutic approaches aimed at restoring ER proteostasis, improving protein-folding capacity, and selectively modulating UPR signaling may represent promising strategies for limiting MASLD progression [23,24,49,50,51].

### 6.3. Oxidative Stress

Oxidative stress is a hallmark of MASLD and arises when reactive oxygen species (ROS) production exceeds the cellular antioxidant defense capacity. Although mitochondria are the primary source of ROS during chronic lipid overload, additional contributors include endoplasmic reticulum stress, peroxisomal fatty acid oxidation, cytochrome P450 enzymes, and activated inflammatory cells. Persistent oxidative stress disrupts redox homeostasis and is a major driver of hepatocellular injury and disease progression [15,17,23,24,49,50,51].

Excessive ROS promote lipid peroxidation, generating highly reactive lipid-derived aldehydes, including malondialdehyde (MDA) and 4-hydroxynonenal (4-HNE), as well as oxidized phospholipids and oxysterols. These reactive lipid peroxidation products not only damage proteins, nucleic acids, and cellular membranes but also act as bioactive signaling molecules that amplify NF-κB activation, NLRP3 inflammasome signaling, and inflammatory cytokine production. As a result, oxidative stress reinforces the interplay between lipid peroxidation, chronic inflammation, and hepatocyte death [20,24,47,51,52].

Beyond promoting inflammation, oxidative stress impairs mitochondrial function, suppresses antioxidant defense mechanisms, and enhances hepatic stellate cell activation through profibrogenic signaling pathways. Crosstalk among ROS, mitochondrial dysfunction, ER stress, and impaired autophagy further accelerates disease progression, contributing to fibrosis and increasing the risk of hepatocellular carcinoma [21,23,24,49,50,51,52].

Despite its central role in MASLD pathogenesis, the clinical benefit of directly targeting oxidative stress remains insufficiently established [23,24,49,52].

### 6.4. Impaired Autophagy and Lipophagy

Autophagy is an evolutionarily conserved lysosomal degradation pathway that maintains cellular homeostasis by removing damaged proteins, dysfunctional organelles, and excess lipid droplets. In the liver, lipophagy, a selective form of autophagy targeting intracellular lipid droplets, plays a pivotal role in regulating hepatic lipid turnover and energy homeostasis. During the early stages of MASLD, autophagy is transiently activated as an adaptive response to nutrient excess and lipid accumulation. However, persistent metabolic stress progressively impairs autophagic flux, promoting hepatocellular lipid accumulation and disease progression (Figure 3) [15,17,23,49,50,51,52].

Lipotoxic lipid species, including saturated fatty acids, ceramides, and free cholesterol, disrupt multiple steps of the autophagic process. Chronic activation of mammalian target of rapamycin complex 1 (mTORC1), lysosomal dysfunction, defective autophagosome maturation, and impaired autophagosome–lysosome fusion collectively reduce autophagic degradation capacity. These alterations are further accompanied by impaired transcription factor EB (TFEB)-dependent lysosomal biogenesis, thereby limiting autophagic capacity. As a result, lipid droplets accumulate within hepatocytes, further enhancing lipotoxicity, oxidative stress, and inflammatory signaling [17,23,49,51,53].

Impaired lipophagy also contributes to mitochondrial dysfunction by limiting the coordinated turnover of lipid droplets and damaged mitochondria. Defective crosstalk between lipophagy and mitophagy promotes reactive oxygen species production, inflammasome activation, and hepatocyte apoptosis, establishing a vicious cycle that accelerates liver injury. Furthermore, impaired autophagy enhances hepatic stellate cell activation and extracellular matrix deposition, linking defective cellular quality control to fibrosis progression [21,23,24,49,53].

Given its role in hepatic metabolic homeostasis, restoration of impaired autophagic flux represents a potential therapeutic strategy for MASLD. Experimental models indicate that enhancing autophagy or lipophagy can reduce steatosis, inflammation, mitochondrial dysfunction, and fibrogenic responses [23,49,53]. However, because autophagy is dynamically regulated and may exert context-, cell-type-, and disease-stage-dependent effects, these preclinical findings cannot yet be directly translated into therapeutic benefit in patients with MASLD.

### 6.5. Mitochondria–ER Crosstalk

Mitochondria and the endoplasmic reticulum (ER) are functionally interconnected through specialized membrane contact sites known as mitochondria-associated ER membranes (MAMs). These dynamic structures coordinate calcium signaling, lipid trafficking, mitochondrial bioenergetics, autophagy, and cellular stress responses, maintaining hepatocellular metabolic homeostasis. In MASLD, chronic lipid overload disrupts MAM integrity and communication, contributing to progressive organelle dysfunction and lipotoxic injury [17,23,24,49,50,51,52,53].

Lipotoxic lipid species impair MAM function by altering membrane composition, disrupting calcium exchange, and promoting mitochondrial calcium overload. These alterations exacerbate mitochondrial dysfunction, increase reactive oxygen species production, and amplify ER stress, establishing a self-reinforcing cycle of metabolic and oxidative injury. Moreover, defective MAM signaling impairs insulin signaling, mitochondrial quality control, and lipid metabolism, further accelerating hepatic steatosis and disease progression [21,23,24,49,51,54].

MAMs also serve as important signaling platforms linking metabolic stress to inflammatory responses. Dysregulated ER–mitochondria communication facilitates activation of the NLRP3 inflammasome, promotes hepatocyte apoptosis, and enhances hepatic stellate cell activation through persistent oxidative and inflammatory signaling. Furthermore, impaired coordination among MAMs, mitophagy, and lipophagy compromises cellular quality control, promoting chronic inflammation and fibrogenesis [21,23,24,49,50,51,52,53,54].

Collectively, MAM dysfunction represents an integrative mechanism linking lipotoxicity with mitochondrial impairment, ER stress, oxidative stress, and defective autophagy in MASLD [23,49,51,54].

Organelle dysfunction thus links metabolic stress to inflammatory and fibrogenic signaling, providing a mechanistic basis for hepatic stellate cell activation, extracellular matrix remodeling, and fibrosis progression discussed in the following chapter.

## 7. Immunometabolic Regulation and Hepatic Inflammation

Lipotoxic lipid accumulation and persistent cellular stress impair hepatocyte function while profoundly remodeling the hepatic immune microenvironment. Through the release of damage-associated molecular patterns (DAMPs), inflammatory cytokines, chemokines, and bioactive lipid mediators, injured hepatocytes establish extensive crosstalk with resident and infiltrating immune cells. These immunometabolic interactions amplify chronic inflammation, promote hepatocellular injury, and drive the transition from simple steatosis to MASH and fibrosis [11,20,21,23,24,47,49,50,51,52,53,54,55].

Recent evidence indicates that hepatic inflammation is not merely a secondary consequence of lipid accumulation but also an active regulator of metabolic homeostasis through dynamic immunometabolic reprogramming of both parenchymal and immune cell populations. Reciprocal interactions among hepatocytes, Kupffer cells, infiltrating monocyte-derived macrophages, neutrophils, lymphocytes, and hepatic stellate cells establish a self-perpetuating inflammatory network that sustains tissue injury and fibrogenesis. The following sections summarize the key cellular and molecular mechanisms through which lipid metabolic dysregulation orchestrates hepatic immune activation and disease progression [20,21,23,24,55].

### 7.1. Kupffer Cells and Hepatic Macrophages

Kupffer cells, the resident macrophages of the liver, together with infiltrating monocyte-derived macrophages, are central regulators of hepatic immunometabolic homeostasis and play a pivotal role in the initiation and progression of MASLD. Under physiological conditions, Kupffer cells maintain immune tolerance by clearing apoptotic cells, pathogens, and cellular debris while supporting tissue homeostasis. During chronic metabolic stress, however, excessive lipid accumulation and hepatocyte injury drive a profound phenotypic shift toward a pro-inflammatory state, promoting persistent hepatic inflammation [20,21,23,24,55].

Lipotoxic lipid species activate Kupffer cells through pattern-recognition receptors such as Toll-like receptor 4 (TLR4)-MyD88-dependent signaling, together with inflammasome-dependent pathways. In parallel, damage-associated molecular patterns (DAMPs) released from injured hepatocytes, including mitochondrial DNA, ATP, and oxidized lipids, further amplify macrophage activation. These stimuli induce the secretion of tumor necrosis factor-α (TNF-α), interleukin-1β (IL-1β), interleukin-6 (IL-6), transforming growth factor-β (TGF-β), and multiple chemokines that recruit circulating monocytes to the liver, sustaining chronic inflammation [21,23,24,47,55].

As MASLD progresses, infiltrating monocyte-derived macrophages increasingly shape the hepatic inflammatory milieu. Interactions between resident Kupffer cells and recruited macrophages generate a highly heterogeneous macrophage population with distinct inflammatory and profibrogenic phenotypes. These macrophages amplify hepatocellular injury and promote hepatic stellate cell activation through the release of TGF-β, platelet-derived growth factor (PDGF), and other profibrotic mediators, establishing a mechanistic link between chronic inflammation and fibrosis progression [23,24,55,56].

Single-cell transcriptomic studies have revealed substantial macrophage heterogeneity in MASLD, identifying disease-associated subsets with distinct lipid-handling, inflammatory, and tissue-remodeling programs. TREM2-positive lipid-associated macrophages are among the best-characterized populations; however, their functional role appears context-dependent and may encompass both adaptive lipid-handling and potentially profibrogenic tissue-remodeling responses. Thus, conventional binary classifications of macrophages as uniformly pro- or anti-inflammatory may oversimplify the dynamic immune landscape of MASLD. Although selective modulation of macrophage immunometabolism is mechanistically attractive, the therapeutic relevance of individual macrophage subsets remains predominantly supported by experimental and single-cell observational evidence and requires further functional and clinical validation [55,56].

### 7.2. Neutrophils and Monocyte Recruitment

Neutrophils and circulating monocytes are rapidly recruited to the liver during MASLD progression and substantially contribute to the amplification of hepatic inflammation. Hepatocyte lipotoxicity, together with Kupffer cell activation, induces the production of chemokines, cytokines, and adhesion molecules that promote leukocyte recruitment. Among these, the CXCL1/CXCL2–CXCR2 axis primarily regulates neutrophil migration, whereas the CCL2–CCR2 pathway is the principal driver of inflammatory monocyte recruitment to the injured liver [20,21,23,24,55,56,57].

Following hepatic infiltration, neutrophils release reactive oxygen species (ROS), myeloperoxidase (MPO), neutrophil elastase, matrix metalloproteinases, and neutrophil extracellular traps (NETs), exacerbating hepatocyte injury and inflammatory signaling. Simultaneously, recruited monocytes differentiate into pro-inflammatory macrophages that further amplify cytokine production, reinforce Kupffer cell activation, and sustain chronic hepatic inflammation. Together, these innate immune responses establish a positive feedback loop that accelerates the transition from steatosis to MASH [21,23,24,55,56,57].

Beyond their direct inflammatory effects, neutrophils and monocyte-derived macrophages actively contribute to fibrogenesis through extensive crosstalk with hepatic stellate cells. Inflammatory mediators, including TNF-α, IL-1β, IL-6, transforming growth factor-β (TGF-β), platelet-derived growth factor (PDGF), and CCL2, stimulate stellate cell activation and extracellular matrix deposition, linking persistent immune cell recruitment to progressive liver fibrosis [21,23,24,55,56,57]. In experimental MASLD models, inhibition of CCR2/CCR5 and CXCR2 signaling reduces inflammatory cell recruitment and liver injury, although clinical translation remains uncertain [23,55,56,57].

### 7.3. T Cells and Adaptive Immunity

Although innate immune activation predominates during the early stages of MASLD, persistent lipotoxicity and hepatic inflammation progressively engage the adaptive immune system. Continuous antigen exposure, hepatocyte injury, and the release of damage-associated molecular patterns (DAMPs) promote T-cell activation and reshape the hepatic immune landscape, driving the transition from simple steatosis to MASH and fibrosis [21,23,24,55,56,57,58].

CD4^+^ T-cell subsets undergo profound functional alterations during disease progression. Increased polarization toward pro-inflammatory T helper 1 (Th1) and T helper 17 (Th17) phenotypes enhances the production of interferon-γ (IFN-γ), tumor necrosis factor-α (TNF-α), and interleukin-17 (IL-17), amplifying macrophage activation, neutrophil recruitment, and hepatic stellate cell activation. In contrast, regulatory T cells (Tregs), which normally maintain immune tolerance and suppress excessive inflammation, exhibit impaired immunoregulatory capacity under chronic metabolic stress, further exacerbating inflammatory responses [21,23,55,58].

CD8^+^ cytotoxic T cells also contribute directly to hepatocellular injury through the release of perforin, granzymes, and pro-inflammatory cytokines. Recent evidence indicates that chronic lipid overload induces metabolic reprogramming and functional exhaustion of hepatic CD8^+^ T cells, impairing immune surveillance while sustaining tissue injury and inflammation. These adaptive immune alterations establish extensive crosstalk with Kupffer cells, monocyte-derived macrophages, and hepatic stellate cells, reinforcing chronic inflammation and fibrogenesis [23,55,56,57,58].

Collectively, dysregulated T-cell responses represent an important component of the immunometabolic network underlying MASLD progression [55,58].

### 7.4. Hepatic Stellate Cell Crosstalk

Persistent hepatic inflammation promotes fibrogenesis through reciprocal crosstalk between immune cells and hepatic stellate cells (HSCs). Activated Kupffer cells, monocyte-derived macrophages, neutrophils, and T cells provide inflammatory and profibrogenic signals that promote HSC activation and extracellular matrix production [21,23,24,55,56,57,58].

Among the major mediators of this intercellular communication, TGF-β and PDGF link inflammatory signaling to HSC activation, proliferation, and fibrogenic responses. Conversely, activated HSCs can influence the hepatic immune microenvironment through cytokine, chemokine, and extracellular matrix-mediated signaling, thereby reinforcing inflammatory–fibrogenic crosstalk [21,23,24,58,59]. The molecular mechanisms and metabolic reprogramming underlying HSC activation are discussed in detail in Section 8.1.

### 7.5. Immunometabolic Crosstalk

The progression of MASLD is driven by a complex bidirectional interplay between metabolic dysfunction and immune activation, collectively referred to as immunometabolic crosstalk. Rather than representing independent processes, lipid metabolic reprogramming and immune responses continuously influence one another through reciprocal signaling among hepatocytes, immune cells, hepatic stellate cells, and non-parenchymal liver cells. This dynamic communication establishes a self-perpetuating inflammatory microenvironment that promotes hepatocellular injury, fibrosis, and disease progression [20,21,23,24,55,56,57,58,59].

Lipotoxic lipid species not only disrupt hepatocyte metabolism but also regulate immune cell phenotype and function. Metabolic stress promotes the release of damage-associated molecular patterns (DAMPs), cytokines, chemokines, extracellular vesicles, and lipid mediators that activate Kupffer cells, recruit monocyte-derived macrophages, stimulate neutrophil infiltration, and modulate adaptive immune responses. In turn, activated immune cells secrete inflammatory mediators that exacerbate mitochondrial dysfunction, endoplasmic reticulum stress, oxidative stress, and hepatocellular lipotoxicity, reinforcing metabolic dysregulation [21,23,24,47,49,50,51,52,53,54,55,56,57,58,59].

Recent studies have demonstrated that metabolic reprogramming critically determines immune cell phenotype and function. Activated macrophages, neutrophils, and effector T cells undergo profound alterations in glycolysis, mitochondrial metabolism, and lipid utilization, which directly influence inflammatory signaling and tissue remodeling. Conversely, changes in hepatic nutrient availability and lipid composition reshape immune cell metabolism, creating a reciprocal feedback loop that sustains chronic inflammation and fibrogenesis [55,56,57,58,59,60]. This emerging concept of immunometabolism provides a mechanistic framework linking metabolic dysfunction with immune-mediated liver injury while may offer new opportunities for therapeutic intervention.

Collectively, immunometabolic crosstalk represents a central pathogenic mechanism underlying MASLD progression. Accordingly, therapeutic strategies that simultaneously target metabolic and immune pathways are increasingly recognized may be more effective than approaches directed at either process alone, emphasizing the need for integrated treatments capable of restoring hepatic metabolic and immune homeostasis [23,55,56,57,58,59,60].

## 8. Lipid Dysregulation and Fibrogenesis

Fibrosis is the principal determinant of long-term clinical outcomes in metabolic dysfunction-associated steatotic liver disease (MASLD) and the strongest histological predictor of liver-related and overall mortality. Although hepatic steatosis and inflammation initiate disease development, persistent lipid metabolic dysregulation ultimately drives excessive extracellular matrix deposition through sustained activation of hepatic stellate cells and extensive intercellular communication within the hepatic microenvironment. Bioactive lipid species, chronic inflammation, oxidative stress, and organelle dysfunction act synergistically to promote fibrogenic signaling, linking metabolic injury to progressive tissue remodeling and irreversible liver damage [11,15,21,23,24,47,48,49,50,51,52,53,54,55,56,57,58,59,60].

The following sections summarize the principal lipid-dependent molecular mechanisms governing hepatic fibrogenesis and discuss their potential as emerging therapeutic targets in MASLD (Table 2).

### 8.1. Hepatic Stellate Cell Activation

Hepatic stellate cell (HSC) activation represents the central cellular event driving liver fibrogenesis in MASLD. Under physiological conditions, quiescent HSCs reside in the space of Disse, where they store vitamin A, regulate extracellular matrix (ECM) turnover, and contribute to hepatic tissue homeostasis. Chronic lipid overload profoundly alters the hepatic microenvironment, triggering the transition of quiescent HSCs into activated myofibroblast-like cells characterized by enhanced proliferation, contractility, and excessive ECM production (Table 2) [21,23,24,55,56,57,58,59,60].

Lipotoxic lipid species directly promote HSC activation in addition to their effects on hepatocyte injury. Saturated fatty acids, free cholesterol, ceramides, and oxidized lipids induce oxidative stress, mitochondrial dysfunction, and inflammatory signaling within stellate cells while increasing their responsiveness to profibrogenic mediators such as transforming growth factor-β (TGF-β) and platelet-derived growth factor (PDGF). In parallel, cytokines and damage-associated molecular patterns (DAMPs) released from injured hepatocytes and activated immune cells further reinforce HSC activation through NF-κB, MAPK, JNK, and SMAD-dependent signaling pathways [21,23,24,47,48,55,56,57,58,59,60].

Activated HSCs undergo extensive metabolic reprogramming characterized by increased aerobic glycolysis (a Warburg-like metabolic phenotype), glutaminolysis, and mitochondrial remodeling, thereby providing the energy and biosynthetic intermediates required for sustained collagen synthesis and myofibroblastic differentiation. Simultaneously, persistent lipid metabolic dysregulation alters intracellular lipid handling, leading to the progressive loss of retinoid-containing lipid droplets, a hallmark of HSC activation. These coordinated metabolic adaptations sustain fibrogenesis while establishing feed-forward mechanisms that perpetuate extracellular matrix deposition and tissue remodeling [21,23,24,59,60,61].

Single-cell transcriptomic studies have further revealed substantial heterogeneity among activated HSC populations, identifying functionally distinct subsets involved in matrix production, immune regulation, and tissue repair [58,59]. This heterogeneity may have important implications for the development of more selective antifibrotic strategies.

Collectively, HSC activation represents a dynamic immunometabolic process rather than a simple consequence of chronic liver injury. The integration of lipid-mediated signaling, metabolic reprogramming, and inflammatory communication places hepatic stellate cells at the center of fibrosis progression and may highlight numerous opportunities for the development of targeted anti-fibrotic therapies for use in MASLD [23,55,56,57,58,59,60,61].

### 8.2. TGF-β Signaling

Transforming growth factor-β (TGF-β) is the master profibrotic cytokine orchestrating hepatic fibrogenesis in MASLD. Although multiple cytokines and growth factors participate in fibrotic remodeling, TGF-β is the principal driver of hepatic stellate cell (HSC) activation, extracellular matrix (ECM) synthesis, and scar formation. Persistent metabolic stress and chronic lipotoxicity sustain TGF-β production by injured hepatocytes, activated Kupffer cells, infiltrating macrophages, neutrophils, and activated HSCs, establishing an autocrine and paracrine signaling network that perpetuates liver fibrosis (Table 2) [21,23,24,55,56,57,58,59,60,61].

Upon binding to transforming growth factor-β receptors (TGFBR1 and TGFBR2), TGF-β activates the canonical SMAD2/3 pathway, leading to the formation of SMAD2/3–SMAD4 transcriptional complexes that translocate to the nucleus and induce the expression of profibrotic genes, including COL1A1, COL3A1, ACTA2 (α-smooth muscle actin), fibronectin, and connective tissue growth factor (CTGF). In parallel, TGF-β activates several non-canonical pathways, including MAPK, JNK, PI3K/Akt, and Rho-associated kinase (ROCK), further promoting HSC proliferation, migration, contractility, and extracellular matrix remodeling [21,23,24,59,61,62].

Lipotoxic lipid species amplify TGF-β signaling through multiple mechanisms. Saturated fatty acids, ceramides, oxidized lipids, and free cholesterol enhance oxidative stress, mitochondrial dysfunction, and inflammatory signaling, increasing TGF-β expression and sensitizing HSCs to profibrotic stimulation. Moreover, reciprocal crosstalk among TGF-β signaling, NF-κB, NLRP3 inflammasome activation, and YAP/TAZ-dependent mechanotransduction pathways establishes a self-reinforcing fibrogenic circuit that promotes progressive extracellular matrix accumulation and tissue stiffening [21,23,24,47,48,55,56,57,58,59,60,61].

Because TGF-β also regulates immune homeostasis, tissue repair, and liver regeneration, global inhibition may result in significant adverse effects, highlighting the need for selective modulation of profibrotic signaling [23,59,60,61,62].

### 8.3. Extracellular Matrix Remodeling

Extracellular matrix (ECM) remodeling is a dynamic and tightly regulated process that maintains liver architecture under physiological conditions. During MASLD progression, however, chronic lipotoxicity and persistent inflammatory signaling disrupt the balance between ECM synthesis and degradation, leading to excessive deposition of fibrillar collagens and scar formation. Activated hepatic stellate cells (HSCs) are the primary source of ECM proteins, producing large amounts of collagen type I and III, fibronectin, laminin, and other matrix components that progressively replace the normal hepatic microenvironment [21,23,24,55,56,57,58,59,60,61,62].

ECM remodeling is governed by the coordinated activity of matrix metalloproteinases (MMPs) and their endogenous inhibitors, the tissue inhibitors of metalloproteininases (TIMPs). Under physiological conditions, MMP-mediated matrix degradation is balanced by TIMP expression, allowing continuous matrix turnover and tissue repair. It should be noted, however, that individual MMP family members exert context-dependent effects, with some enzymes facilitating matrix resolution whereas others contribute to inflammatory remodeling and fibrosis progression. In MASLD, persistent TGF-β signaling and inflammatory cytokines markedly increase TIMP expression while suppressing matrix degradation, resulting in excessive ECM accumulation and stabilization of fibrotic tissue [21,23,24,59,61,62,63].

Beyond providing structural support, the remodeled ECM actively regulates cellular behavior through biochemical and mechanical signaling. Progressive matrix stiffening alters integrin-mediated cell–matrix interactions and activates mechanosensitive pathways, including focal adhesion kinase (FAK), Yes-associated protein (YAP), and transcriptional coactivator with PDZ-binding motif (TAZ), further promoting hepatic stellate cell activation, inflammatory signaling, and fibrogenesis. Consequently, ECM remodeling establishes a feed-forward profibrotic loop in which matrix deposition perpetuates fibrotic progression (Table 2) [23,59,61,62,63].

Recent advances in matrix biology have demonstrated that the composition and organization of the hepatic ECM influence immune cell recruitment, angiogenesis, and tissue regeneration in addition to fibrosis. These findings identify ECM remodeling as an active regulator of the hepatic microenvironment rather than a passive consequence of chronic liver injury.

These findings establish ECM remodeling as an active component of the fibrogenic microenvironment rather than merely a structural consequence of chronic liver injury [23,59,60,61,62,63].

### 8.4. Progression Toward Cirrhosis

Progressive fibrosis ultimately culminates in cirrhosis, the end stage of chronic liver injury and the strongest predictor of liver-related morbidity and mortality in MASLD. Although fibrosis initially represents an adaptive wound-healing response, persistent lipid metabolic dysregulation, chronic inflammation, and sustained hepatic stellate cell activation gradually transform this reparative process into irreversible architectural remodeling. Excessive extracellular matrix deposition disrupts the normal hepatic lobular structure, leading to fibrous septa formation, regenerative nodule development, and distortion of the hepatic vascular network [21,23,24,55,56,57,58,59,60,61,62,63].

The transition from advanced fibrosis to cirrhosis is driven by sustained immunometabolic dysfunction. Persistent hepatocyte lipotoxicity, mitochondrial dysfunction, oxidative stress, endoplasmic reticulum stress, and chronic activation of Kupffer cells, infiltrating macrophages, neutrophils, and adaptive immune cells maintain a profibrotic microenvironment that stimulates extracellular matrix production while limiting matrix degradation. In parallel, progressive liver sinusoidal endothelial cell (LSEC) capillarization, endothelial dysfunction, and increasing matrix stiffness further amplify hepatic stellate cell activation through mechanotransduction pathways, establishing a self-perpetuating cycle of fibrosis progression (Table 2) [21,23,24,47,49,50,51,52,53,54,55,56,57,58,59,60,61,62,63,64].

Beyond structural remodeling, cirrhosis profoundly alters hepatic function. Progressive replacement of functional parenchyma by fibrotic tissue impairs hepatic metabolism, bile acid homeostasis, protein synthesis, detoxification, and immune regulation while increasing intrahepatic vascular resistance and portal hypertension. These alterations substantially increase the risk of hepatic decompensation, liver failure, and hepatocellular carcinoma, highlighting cirrhosis as a systemic consequence of persistent metabolic and inflammatory injury rather than merely excessive collagen deposition [2,7,8,21,24,55,56,57,58,59,60,61,62,63,64].

Recent evidence suggests that liver fibrosis remains partially reversible during its early stages if the underlying metabolic and inflammatory drivers are effectively controlled. However, once extensive architectural distortion and regenerative nodules are established, complete restoration of normal hepatic structure becomes increasingly unlikely. These findings emphasize the importance of early therapeutic intervention aimed at correcting lipid metabolic dysregulation, suppressing chronic inflammation, and interrupting fibrogenic signaling before irreversible cirrhotic remodeling develops [15,23,24,59,60,61,62,63,64]. Collectively, these observations provide a strong mechanistic rationale for the development of targeted antifibrotic therapies capable of modifying disease progression in MASLD.

## 9. Lipidomics and Precision Hepatology

Recent advances in high-resolution lipidomics have fundamentally transformed our understanding of MASLD by enabling comprehensive characterization of hepatic and circulating lipid species beyond conventional lipid measurements. Rather than viewing steatosis as a simple consequence of triglyceride accumulation, lipidomic technologies have revealed disease-specific lipid signatures associated with hepatocellular lipotoxicity, inflammation, fibrosis, and clinical outcomes. These advances have established lipidomics as a key translational platform linking molecular mechanisms to clinical decision-making [25,26,27,28,29,44,45,46,47,48,60,64].

The integration of lipidomics with genomics, transcriptomics, proteomics, metabolomics, and advanced computational approaches has accelerated the development of precision hepatology. Multi-omics analyses have identified distinct molecular endotypes of MASLD that differ in metabolic dysfunction, inflammatory activity, fibrogenic potential, and therapeutic responsiveness. Consequently, comprehensive lipid profiling is increasingly recognized as a promising strategy for improving disease stratification, non-invasive diagnosis, prognostic assessment, and individualized therapeutic selection [25,26,27,28,29,60,64].

The following sections summarize the emerging role of lipidomics in biomarker discovery, patient stratification, and precision medicine while highlighting its potential to facilitate personalized management of MASLD and accelerate the translation of mechanistic insights into clinical practice (Table 3) [60,64].

### 9.1. Lipidomic Biomarkers

Lipidomic profiling has identified distinct molecular signatures reflecting the metabolic heterogeneity of MASLD. Unlike conventional lipid measurements, comprehensive lipidomic analyses simultaneously quantify numerous bioactive lipid species associated with different pathogenic processes and disease stages [25,26,27,28,29,44,45,46,47,48,60,65].

Candidate lipidomic biomarkers include ceramides, diacylglycerols, oxidized phospholipids, lysophospholipids, bile acid metabolites, and specific triglyceride species. Alterations in these lipid classes have been associated with hepatic insulin resistance, lipotoxicity, MASH, and fibrosis, supporting their potential relevance as molecular biomarkers [20,25,26,27,28,29,44,45,46,47,48,60,65]. Importantly, currently proposed lipidomic signatures should be regarded as investigational rather than clinically validated biomarkers, and their incremental clinical value over established non-invasive approaches remains to be demonstrated [65,66].

The integration of lipidomic biomarkers with clinical variables, imaging modalities, and other omics technologies may improve patient stratification and prognostic assessment. However, several barriers currently limit the translation of lipidomic signatures into routine clinical practice. Differences in sample collection and processing, analytical platforms, lipid extraction procedures, data normalization, batch effects, and other preanalytical and patient-related factors, including fasting status, medication use, and relevant clinical confounders, should be considered when developing and validating lipidomic biomarkers because they may impair the reproducibility and comparability of reported lipid signatures [66,67]. Harmonized analytical workflows, standardized reference materials, and consensus approaches to data processing and interpretation are therefore required to improve cross-platform and cross-laboratory comparability [66,67]. In addition, the cost and technical complexity of high-resolution lipidomic platforms remain practical barriers to widespread clinical implementation, and their incremental clinical value and cost-effectiveness relative to established non-invasive tests require further evaluation [66]. Finally, proposed lipidomic signatures require independent external validation in large, prospective, multicenter cohorts encompassing diverse patient populations before they can be considered sufficiently robust for routine clinical implementation [65,66].

### 9.2. Precision Medicine

The growing understanding of lipid metabolic reprogramming has accelerated the transition from conventional disease classification to precision hepatology. Rather than viewing MASLD as a single clinical entity, increasing evidence indicates substantial interindividual variability in lipid metabolism, inflammatory responses, fibrogenic activity, and therapeutic responsiveness. Consequently, integrating lipidomic data with clinical, genetic, transcriptomic, proteomic, and metabolomic information may enable a more comprehensive characterization of disease heterogeneity and molecular endotypes [25,26,27,28,29,60,65,68].

Lipidomic profiling may help identify molecular endotypes associated with distinct pathogenic mechanisms and clinical outcomes. Combined with genetic risk variants and emerging polygenic risk scores, including PNPLA3, TM6SF2, MBOAT7, and HSD17B13, integrated molecular signatures may improve stratification according to disease progression and therapeutic responsiveness [25,26,27,28,29,60,65,68].

Precision medicine may also offer opportunities to optimize therapeutic decision-making. Molecular profiling may additionally serve as a companion diagnostic strategy by identifying patients most likely to benefit from therapies targeting de novo lipogenesis, bile acid signaling, nuclear receptors, lipid oxidation, or inflammatory pathways.

### 9.3. Diagnostic and Prognostic Applications

Lipidomic profiling may complement conventional biochemical markers and imaging modalities by providing molecular information relevant to disease activity and progression. This is particularly relevant given the limitations of liver biopsy for repeated longitudinal assessment [25,26,27,28,29,60,65,66,67].

Several exploratory studies suggest that circulating lipid may help distinguish steatosis from MASH and identify patients at increased risk of advanced fibrosis. Composite multimodal biomarker panels incorporating different lipid classes may provide complementary molecular information; however, their incremental diagnostic value over established non-invasive approaches remains to be demonstrated.

Beyond diagnosis, disease-associated lipid signatures have shown potential prognostic associations with fibrosis progression and adverse liver-related outcomes. Their potential use for longitudinal disease monitoring, treatment-response assessment, and as pharmacodynamic biomarkers is of considerable interest but remains investigational and requires prospective validation [60,65,66,67,68,69].

Despite these advances, the clinical utility of lipidomic and integrated multi-omics approaches remains to be established. Their diagnostic and prognostic performance requires prospective validation, methodological harmonization, and demonstration of incremental clinical value over established non-invasive approaches before routine clinical implementation [65,66,67].

## 10. Emerging Therapeutic Opportunities

Growing insight into the molecular mechanisms underlying metabolic dysfunction-associated steatotic liver disease (MASLD) has shifted therapeutic development from reducing hepatic steatosis alone toward targeting the interconnected metabolic, lipotoxic, cellular stress, inflammatory, and fibrogenic pathways that drive disease progression [23,31,70,71]. Current therapeutic strategies differ substantially in translational maturity, ranging from predominantly preclinical approaches to clinically validated mechanism-based therapies. The present section focuses primarily on therapies targeting the lipid-metabolic and lipid-associated pathogenic mechanisms discussed in this review rather than providing a comprehensive overview of MASH pharmacotherapy. Nevertheless, semaglutide (Wegovy), which received FDA accelerated approval in August 2025 for adults with noncirrhotic MASH and moderate-to-advanced fibrosis, is included because of its current clinical relevance [72].

The principal therapeutic targets, evidence levels and regulatory status, limitations, and potential roles in combination strategies are summarized in Table 4.

### 10.1. Targeting De Novo Lipogenesis

Enhanced hepatic de novo lipogenesis (DNL) represents an attractive therapeutic target because it contributes to both triglyceride accumulation and the generation of lipotoxic lipid species [15,23,32]. Pharmacological inhibition of acetyl-CoA carboxylase (ACC), fatty acid synthase (FASN), and stearoyl-CoA desaturase-1 (SCD1) has therefore been investigated as a strategy to reduce hepatic lipid accumulation and lipotoxicity [23,31,71]. However, clinical translation remains limited by target-specific adverse metabolic effects and variable efficacy. In particular, ACC inhibition may increase circulating triglycerides, whereas the FASN inhibitor denifanstat has demonstrated improvements in MASH activity and fibrosis-related histological endpoints in a phase IIb trial [71,73]. These findings support continued evaluation of DNL-directed therapies, while emphasizing the need to balance efficacy with metabolic safety.

### 10.2. Nuclear Receptor-Directed Therapies

Nuclear receptors represent some of the most clinically advanced molecular targets in MASLD because they coordinately regulate lipid metabolism, insulin sensitivity, inflammation, and fibrogenesis [18,19,31]. The strongest clinical validation has been achieved with the selective thyroid hormone receptor-β (THR-β) agonist resmetirom, which significantly improved MASH resolution and fibrosis in a phase III trial and became the first FDA-approved pharmacological therapy for adults with noncirrhotic MASH and moderate-to-advanced fibrosis [70,71]. The pan-PPAR agonist lanifibranor has also demonstrated improvements in steatohepatitis activity and fibrosis-related outcomes [41], whereas FXR agonists have shown metabolic and histological activity but their development has been constrained by adverse effects, including pruritus and unfavorable changes in serum lipid profiles [19,42,71]. These findings illustrate both the potential and the limitations of targeting pleiotropic metabolic regulators and emphasize the importance of balancing efficacy with long-term safety.

### 10.3. Targeting Lipotoxicity and Ceramide Metabolism

Ceramide metabolism represents a mechanistically attractive but less clinically mature therapeutic target. Experimental modulation of serine palmitoyltransferase (SPT), dihydroceramide desaturase-1 (DES1), and other enzymes involved in ceramide metabolism has been associated with improvements in insulin signaling, mitochondrial function, inflammation, and hepatic lipid accumulation in preclinical models [21,44,45]. However, sphingolipids also perform essential structural and signaling functions, raising concerns regarding systemic inhibition and emphasizing the need for tissue-, enzyme-, and lipid-species-selective approaches [21,44,45]. Consequently, ceramide-directed therapies remain predominantly preclinical, and their efficacy and safety in human MASLD require clinical validation [45].

### 10.4. Targeting Mitochondrial and Endoplasmic Reticulum Stress

Mitochondrial dysfunction, oxidative stress, and maladaptive endoplasmic reticulum (ER) stress responses contribute to hepatocellular injury and provide additional therapeutic opportunities in MASLD [24,49,50,51,52]. Preclinical approaches aimed at restoring mitochondrial function, improving mitochondrial quality control and mitophagy, reducing oxidative injury, or modulating ER stress responses have shown hepatoprotective potential [49,50,51,52,53,54]. Nevertheless, these pathways are integral to normal cellular homeostasis, and their context- and disease-stage-dependent functions complicate pharmacological targeting. Evidence therefore remains predominantly preclinical, and selective modulation rather than global pathway inhibition will likely be required for successful clinical translation [49,50,51,52,53,54].

### 10.5. Anti-Inflammatory and Antifibrotic Strategies

Lipid-driven inflammation and hepatic stellate cell activation provide complementary therapeutic targets in progressive MASH. Experimental modulation of NF-κB and NLRP3 signaling can attenuate inflammatory activation, hepatocellular injury, and fibrogenic responses, although broad suppression of immune signaling may compromise physiological immune functions [21,23,24,55,60]. Similarly, direct targeting of TGF-β/SMAD signaling and other pathways involved in hepatic stellate cell activation and extracellular matrix remodeling has demonstrated antifibrotic potential in experimental models, but systemic inhibition remains challenging because these pathways also regulate tissue homeostasis, immune regulation, and repair [59,61,62,63]. These limitations favor selective pathway modulation and strategies that simultaneously reduce the upstream metabolic and inflammatory stimuli driving fibrogenesis [23,31,59,71].

### 10.6. Single-Pathway Versus Combination Therapy

The clinical efficacy of resmetirom demonstrates that targeting a single biologically relevant pathway can provide meaningful therapeutic benefit in appropriately selected patients [70]. Nevertheless, MASLD is driven by interconnected abnormalities in lipid metabolism, organelle function, inflammation, hepatocellular injury, and fibrogenesis, and modulation of a single pathway may not fully address this biological complexity [23,31,71]. This provides a strong rationale for mechanism-based combinations targeting complementary pathogenic processes [31,71,74].

Combination therapy, however, should not be assumed to be intrinsically superior to monotherapy. Potential additive or synergistic efficacy must be balanced against increased treatment complexity, adverse effects, drug–drug interactions, and cost. Moreover, the molecular heterogeneity of MASLD suggests that optimal combinations may differ according to disease stage and dominant pathogenic phenotype [65,68,74]. Future clinical trials should therefore establish which mechanistically complementary combinations provide clinically meaningful benefit and identify the molecular and clinical phenotypes most likely to respond to specific therapeutic strategies [65,71,74].

## 11. Conclusions and Future Perspectives

MASLD is increasingly recognized as a multifactorial metabolic disorder in which disease progression is driven not merely by excessive hepatic lipid accumulation but by profound alterations in lipid metabolic reprogramming and lipid-mediated signaling. Rather than serving as passive storage molecules, bioactive lipids—including saturated fatty acids, ceramides, diacylglycerols, oxidized lipids, and free cholesterol—orchestrate interconnected signaling networks that regulate insulin resistance, mitochondrial dysfunction, oxidative stress, inflammation, and fibrogenesis, thereby promoting the transition from simple steatosis to MASH, advanced fibrosis, and cirrhosis [11,12,13,14,15,16,17,18,19,20,21,22,23,24].

Hepatic lipid metabolic reprogramming is a dynamic process involving coordinated alterations in lipid synthesis, oxidation, transport, storage, and signaling that interact with organelle dysfunction and immune activation. This integrated framework helps explain why therapeutic strategies targeting individual pathological processes have often demonstrated only limited clinical efficacy [15,16,17,18,19,20,21,22,23,24,49,50,51,52,53].

Recent advances in lipidomics and multi-omics technologies have further expanded our understanding of disease heterogeneity and identified novel molecular signatures associated with disease severity, fibrosis progression, and therapeutic responsiveness. Integrating lipidomic data with transcriptomic, proteomic, and metabolomic profiling is expected to improve patient stratification, facilitate biomarker discovery, and support the implementation of precision hepatology through more accurate diagnosis, prognostic assessment, and treatment monitoring [25,26,27,28,29,60,65,66,67,68,69].

These mechanistic insights may accelerate the development of targeted therapeutic strategies. Current pharmacological approaches increasingly focus on restoring hepatic metabolic homeostasis through modulation of de novo lipogenesis, nuclear receptor signaling, lipotoxicity, inflammatory pathways, and fibrogenesis rather than solely reducing hepatic fat accumulation. The recent clinical success of mechanism-based therapies, particularly thyroid hormone receptor-β agonists, together with the continued development of combination treatment strategies, highlights the translational potential of simultaneously targeting multiple interconnected pathogenic pathways [15,18,19,23,31,70,71].

Despite substantial progress, several key scientific barriers remain before mechanistic insights into lipid metabolic reprogramming can be translated into reliable biomarkers and truly disease-modifying therapies. A major unresolved challenge is to distinguish lipid alterations that are causal drivers of disease progression from those that primarily reflect downstream metabolic injury, particularly given the species-, compartment-, cell-type-, and disease-stage-specific effects of bioactive lipids. In parallel, the marked molecular heterogeneity of MASLD complicates the identification of universally applicable biomarkers and therapeutic targets. Future research should therefore prioritize longitudinal and mechanistically informed studies that establish causal relationships between lipid signatures and disease progression, validate reproducible biomarkers across diverse populations and analytical platforms, and determine whether molecular endotypes can reliably predict clinical outcomes and therapeutic response [25,26,27,28,29,60,65,66,67,68,69]. Ultimately, integrating validated lipidomic and multi-omic signatures with clinical phenotyping will be essential for translating molecular heterogeneity into actionable patient stratification and mechanism-based, potentially combinatorial therapeutic strategies [65,68,71,72,73,74].

An emerging additional consideration is the potential spleen–liver immunometabolic axis, through which splenic immune-cell trafficking and bioactive lipid signaling may contribute to hepatic inflammation and fibrogenesis, although its mechanistic and clinical relevance in human MASLD remains to be established [75].

In conclusion, the transition from a steatosis-centered concept of MASLD towards a comprehensive understanding of lipid metabolic reprogramming and lipid signaling networks represents a major paradigm shift in hepatology. Continued integration of mechanistic research, high-resolution molecular profiling, and targeted therapeutic development may enable biomarker-guided, mechanism-based strategies capable of improving patient stratification and modifying disease progression in MASLD.

## Figures and Tables

**Table 1:** Major lipotoxic lipid species and their pathogenic effects in MASLD.

Lipid Species	Primary Source/Metabolic Origin	Major Pathogenic Mechanisms	Principal Biological Consequences	References
Saturated fatty acids (SFAs) (e.g., palmitate)	Increased adipose tissue lipolysis, enhanced de novo lipogenesis, excessive dietary intake	ER stress, mitochondrial dysfunction, oxidative stress, activation of JNK, NF-κB and NLRP3 pathways	Hepatocyte injury, apoptosis, inflammation, initiation of lipotoxicity	[11,12,13,15,23,43]
Ceramides	De novo sphingolipid synthesis, sphingomyelin hydrolysis, salvage pathway	Inhibition of Akt signaling, activation of PP2A, mitochondrial dysfunction, ROS generation, ER stress	Insulin resistance, inflammation, apoptosis, fibrosis progression	[11,13,20,21,23,44]
Complex sphingolipids (sphingomyelins, S1P, glycosphingolipids)	Ceramide metabolism via sphingosine kinase and sphingomyelin pathways	Regulation of S1P receptor signaling, immune cell activation, inflammatory and fibrogenic responses	Modulation of inflammation, hepatic stellate cell activation, fibrosis	[20,25,26,29,44,45]
Diacylglycerols (DAGs)	Excess fatty acid influx, enhanced de novo lipogenesis, impaired triglyceride turnover	Activation of PKCε and related PKC isoforms, impaired insulin receptor signaling	Hepatic insulin resistance, metabolic dysfunction, altered glucose homeostasis	[3,11,15,23,46]
Oxidized lipids (OxPLs, oxysterols, oxidized lipoproteins)	Lipid peroxidation during oxidative stress	Activation of pattern-recognition receptors, DAMP signaling, cytokine production, mitochondrial injury	Chronic inflammation, immune activation, disease progression	[20,24,25,26,27,28,29,47]
Free cholesterol	Increased cholesterol synthesis and uptake, impaired esterification and biliary excretion	Membrane destabilization, ER and mitochondrial stress, cholesterol crystal formation, NLRP3 activation	Hepatocyte death, Kupffer cell activation, fibrosis progression	[15,21,23,24,48]

**Table 2:** Lipid-driven fibrogenic mechanisms and their consequences in MASLD.

Fibrogenic Process	Key Cellular Drivers	Major Molecular Mechanisms	Pathogenic Consequences	References
Hepatic stellate cell activation	Quiescent HSCs, injured hepatocytes, Kupffer cells, macrophages	Lipotoxic lipids, DAMPs, oxidative stress, NF-κB, MAPK, JNK, SMAD signaling	Myofibroblast-like transformation, proliferation, contractility, ECM production	[21,23,24,47,48,55,56,57,58,59,60,61]
TGF-β signaling	Activated HSCs, Kupffer cells, infiltrating macrophages, injured hepatocytes	TGFBR1/2 activation, SMAD2/3–SMAD4 signaling, MAPK, PI3K/Akt, ROCK pathways	COL1A1, COL3A1, ACTA2, CTGF and fibronectin expression; sustained profibrotic phenotype	[21,23,24,55,56,57,58,59,60,61,62]
Extracellular matrix remodeling	Activated HSCs, portal fibroblasts, macrophages, liver sinusoidal endothelial cells	Increased collagen I/III, fibronectin and laminin synthesis; ↑ TIMPs; ↓ MMP-mediated matrix degradation	ECM accumulation, collagen cross-linking, matrix stiffening, scar formation	[21,23,24,59,60,61,62,63]
Mechanotransduction and matrix stiffening	Activated HSCs, ECM components, endothelial cells	Integrin/FAK signaling, YAP/TAZ activation, altered cell–matrix interactions	Positive feedback sustaining HSC activation and fibrogenesis	[23,59,61,62,63]
Progression toward cirrhosis	Activated HSCs, immune cells, endothelial cells, hepatocytes	Persistent lipotoxicity, chronic inflammation, ECM deposition, sinusoidal capillarization, vascular remodeling	Fibrous septa, regenerative nodules, portal hypertension, impaired liver function, HCC risk	[2,7,8,21,24,55,56,57,58,59,60,61,62,63]

**Table 3:** Clinical applications of lipidomics in precision hepatology for MASLD.

Clinical Application	Lipidomic Targets	Potential Clinical Utility	Current Limitations	Representative References
Early disease detection	Ceramides, diacylglycerols (DAGs), phosphatidylcholines (PCs), lysophosphatidylcholines (LPCs)	Identification of early metabolic abnormalities before advanced liver injury	Inter-platform variability; lack of standardized cut-off values	[25,26,27,28,29,44,45,46,47,48,60,65]
Differentiation of steatosis and MASH	Ceramide panels, oxidized phospholipids, oxylipins, bile acid metabolites	Improved discrimination between simple steatosis and steatohepatitis compared with single biomarkers	Requires validation in large prospective cohorts	[25,26,27,28,29,44,45,46,47,48,60,65]
Fibrosis risk stratification	Ceramides, DAGs, bile acids, phospholipid signatures	Identification of patients at risk for advanced fibrosis; complementary to FIB-4, ELF score and elastography	Limited clinical standardization and reimbursement	[60,65,66,67]
Prognostic assessment	Composite lipidomic signatures integrating multiple lipid classes	Prediction of fibrosis progression, cirrhosis and hepatocellular carcinoma risk	Long-term prospective outcome studies remain limited	[60,65,66,67]
Therapeutic response monitoring	Dynamic changes in circulating lipid profiles during treatment	Evaluation of pharmacodynamic responses and treatment efficacy	Biomarker qualification is still ongoing	[60,65,66]
Precision hepatology/ molecular endotyping	Integrated lipidomic, genomic, transcriptomic and clinical datasets	Patient stratification, molecular endotyping and individualized therapeutic selection	Multi-omics integration, bioinformatics infrastructure and regulatory validation are required	[60,65,66]

**Table 4:** Emerging therapeutic opportunities in MASLD.

Therapeutic Domain	Targets/Strategies	Evidence	Potential Benefit	Key Limitations	Combination Rationale	References.
De novo lipogenesis	ACC; FASN (denifanstat); SCD1	Preclinical–phase IIb	Reduced DNL/lipotoxicity; histological benefit with denifanstat	Hypertriglyceridemia with ACC inhibition; long-term safety	Complementary to anti-inflammatory/antifibrotic therapy	[15,23,31,32,71,73]
Nuclear receptors	THR-β (resmetirom); PPAR (lanifibranor); FXR	FDA-approved Investigational—clinical development	MASH resolution and/or fibrosis improvement	Pruritus/lipid changes with FXR agonists; long-term safety	Potential metabolic backbone for combinations	[18,19,31,41,42,70,71]
Ceramide/sphingolipid metabolism	SPT; DES1; CerS; S1P/S1PR	Predominantly preclinical	Improved insulin signaling, inflammation, and steatosis	Essential physiological functions; selectivity required	May complement metabolic/anti-inflammatory therapy	[21,44,45,71]
Mitochondrial / ER stress	Mitochondrial function; mitophagy; oxidative/ER stress	Mainly preclinical	Reduced cellular stress and hepatocellular injury	Context-dependent homeostatic functions	May limit stress amplification	[24,49,50,51,52,53,54]
Anti-inflammatory	NF-κB; NLRP3; immunometabolic pathways	Predominantly preclinical	Reduced inflammation and injury	Risk of broad immune suppression	Combine with upstream metabolic targeting	[21,23,24,55,56,57,58,59,60]
Antifibrotic	HSC; TGF-β/SMAD; ECM remodeling	Largely preclinical	Reduced fibrogenic signaling	Homeostatic/repair functions limit systemic inhibition	Complement metabolic/anti-inflammatory therapy	[59,61,62,63,71]
Combination/precision therapy	Complementary targets; phenotype-guided selection	Promising; validation needed	Potential broader disease modification	Complexity, safety, interactions, cost	Tailor to disease stage and phenotype	[65,68,71,74]

## Data Availability

No new data were created or analyzed in this study. Data sharing is not applicable to this article.

## Abbreviations

ACC	Acetyl-CoA Carboxylase
ACTA2	Actin Alpha 2, Smooth Muscle
Akt (PKB)	Protein Kinase B
AMPK	AMP-Activated Protein Kinase
AP-1	Activator Protein-1
ApoB100	Apolipoprotein B100
ASC	Apoptosis-Associated Speck-Like Protein Containing a Caspase Recruitment Domain
ATF6	Activating Transcription Factor 6
ATGL	Adipose Triglyceride Lipase
ATP	Adenosine Triphosphate
β-oxidation	Fatty Acid Beta-Oxidation
CerS	Ceramide Synthase
CHOP	C/EBP Homologous Protein
ChREBP	Carbohydrate-Responsive Element-Binding Protein
COL1A1	Collagen Type I Alpha 1 Chain
COL3A1	Collagen Type III Alpha 1 Chain
CPT1A	Carnitine Palmitoyltransferase 1A
CTGF	Connective Tissue Growth Factor
CYP7A1	Cholesterol 7 Alpha-Hydroxylase
DAG	Diacylglycerol
DAMP	Damage-Associated Molecular Pattern
DES1	Dihydroceramide Desaturase-1
DNL	De Novo Lipogenesis
ECM	Extracellular Matrix
ELF	Enhanced Liver Fibrosis
ER	Endoplasmic Reticulum
ERK	Extracellular Signal-Regulated Kinase
ETC	Electron Transport Chain
FAK:	Focal Adhesion Kinase
FASN	Fatty Acid Synthase
FATP	Fatty Acid Transport Protein
FDA	Food and Drug Administration
FGF19:	Fibroblast Growth Factor 19
FFA:	Free Fatty Acid
FIB-4	Fibrosis-4 Index
FXR	Farnesoid X Receptor
GPAT	Glycerol-3-Phosphate Acyltransferase
HCC	Hepatocellular Carcinoma
HDL	High-Density Lipoprotein
HSC	Hepatic Stellate Cell
HSL	Hormone-Sensitive Lipase
IL	Interleukin
IκB	Inhibitor of Nuclear Factor-κB
IRE1α	Inositol-Requiring Enzyme 1 Alpha
IRS-1	Insulin Receptor Substrate-1
JNK	c-Jun N-Terminal Kinase
LDL	Low-Density Lipoprotein
LPC	Lysophosphatidylcholine
LPK	Liver-Type Pyruvate Kinase
LXR	Liver X Receptor
MAM	Mitochondria-Associated Membrane
MAPK	Mitogen-Activated Protein Kinase
MASH	Metabolic Dysfunction-Associated Steatohepatitis
MASLD	Metabolic Dysfunction-Associated Steatotic Liver Disease
MCP-1	Monocyte Chemoattractant Protein-1
MDA	Malondialdehyde
MMP	Matrix Metalloproteinase
MTP	Microsomal Triglyceride Transfer Protein
mTOR	Mechanistic Target of Rapamycin
mTORC1	Mechanistic Target of Rapamycin Complex 1
mtDNA	Mitochondrial DNA
MyD88	Myeloid Differentiation Primary Response Protein 88
NAFLD	Nonalcoholic Fatty Liver Disease
NF-κB	Nuclear Factor Kappa B
NLRP3	NOD-, LRR-, and Pyrin Domain-Containing Protein 3
OSES	Oxidation-Specific Epitopes
OxPL	Oxidized Phospholipid
PC	Phosphatidylcholine
PERK	Protein Kinase RNA-like Endoplasmic Reticulum Kinase
PI3K	Phosphoinositide 3-Kinase
PKC	Protein Kinase C
PP2A	Protein Phosphatase 2A
PPAR	Peroxisome Proliferator-Activated Receptor
ROS	Reactive Oxygen Species
ROCK	Rho-Associated Protein Kinase
RXR	Retinoid X Receptor
S1P	Sphingosine-1-Phosphate
S1PR	Sphingosine-1-Phosphate Receptor
SCD1	Stearoyl-CoA Desaturase-1
SFA	Saturated Fatty Acid
SHP	Small Heterodimer Partner
SMAD	Suppressor of Mothers Against Decapentaplegic
SphK	Sphingosine Kinase
SPT	Serine Palmitoyltransferase
SREBP-1c	Sterol Regulatory Element-Binding Protein-1c
T2DM	Type 2 Diabetes Mellitus
TGF-β	Transforming Growth Factor Beta
TGFBR	Transforming Growth Factor Beta Receptor
THR-β	Thyroid Hormone Receptor Beta
TIMP	Tissue Inhibitor of Metalloproteinases
TLR4	Toll-Like Receptor 4
TNF-α	Tumor Necrosis Factor Alpha
UPR	Unfolded Protein Response
VLDL	Very-Low-Density Lipoprotein
YAP/TAZ	Yes-Associated Protein/Transcriptional Coactivator with PDZ-Binding Motif
